# Inhibitory effect of thiamine salts on steel corrosion in an acidic environment: an experimental and theoretical approach

**DOI:** 10.1039/d6ra02411d

**Published:** 2026-05-14

**Authors:** Dinh Quy Huong, Nguyen Phuc Quynh Ly, Pham Dinh Tu Tai, Dinh Tuan, Nguyen Minh Tam, Le Quoc Thang, Pham Cam Nam

**Affiliations:** a Department of Chemistry, University of Education, Hue University Hue City Vietnam dqhuong@hueuni.edu.vn; b Department of Planning, Finance and Facilities Management, Hue University Hue City Vietnam; c Faculty of Pharmacy, Nam Can Tho University 168 Nguyen Van Cu Ext Can Tho Vietnam; d Faculty of Basic Sciences & Interdisciplinary Research Transfer and Innovation Center, University of Phan Thiet 225 Nguyen Thong Phu Thuy Lam Dong Vietnam; e Faculty of Chemical Engineering & Strategic Materials & Advanced Research Team – DUT (SMART-DUT), The University of Danang, University of Science and Technology Danang 550000 Vietnam

## Abstract

The effectiveness of thiamine hydrochloride and thiamine pyrophosphate as corrosion inhibitors for steel in 1.0 M hydrochloric acid medium was assessed through electrochemical techniques supported by theoretical calculations. The results indicate that both compounds exhibit significant corrosion inhibition efficiency in HCl, with thiamine hydrochloride providing better protection for the steel surface than thiamine pyrophosphate. According to the results obtained from electrochemical impedance spectroscopy, at a concentration of 5 × 10^−3^ M, the inhibition performance of thiamine hydrochloride reached 87.87%, whereas thiamine pyrophosphate exhibited an efficiency of 85.28%. Potentiodynamic polarization measurements reveal that both thiamine hydrochloride and thiamine pyrophosphate act as mixed-type inhibitors. The inhibition ability of the compounds tends to decrease with increasing temperature, indicating a reduction in the stability of the adsorbed protective layer on the steel surface at elevated temperatures. Adsorption isotherm analysis suggests that the adsorption behavior of thiamine hydrochloride and thiamine pyrophosphate molecules on the steel surface is well described by the Langmuir adsorption model. Surface morphology observations show that the steel sample subjected to immersion in a thiamine hydrochloride solution exhibits significantly less surface damage compared with the sample in the presence of thiamine pyrophosphate. Theoretical analysis and molecular dynamics simulations further indicate that thiamine hydrochloride has a stronger interaction and better surface coverage on the Fe surface than thiamine pyrophosphate. These findings are consistent with the experimental data, confirming that thiamine hydrochloride outperforms thiamine pyrophosphate in mitigating the steel corrosion process in hydrochloric acid medium.

## Abstract 2

Thiamine hydrochloride outperforms thiamine pyrophosphate as a corrosion inhibitor due to its stronger adsorption on the steel surface.

## Graphical abstract

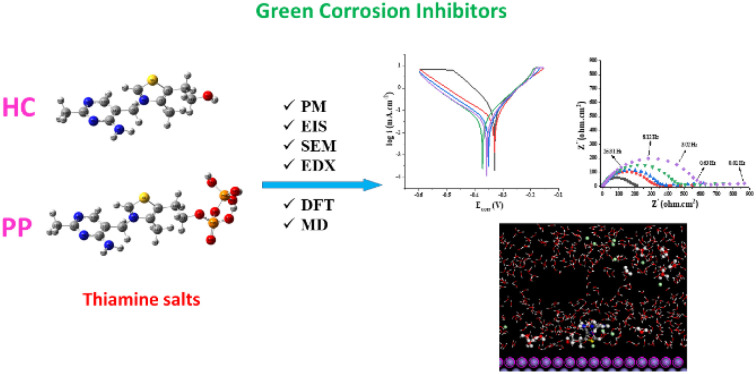

## 1 Introduction

Steel is an important metallic material widely used in fields such as construction, transportation, energy, chemical industries, and mechanical engineering due to its high mechanical strength and cost-effectiveness.^1^ Nonetheless, in aggressive environments (acids, alkalis, saline solutions, humid media, or environments containing Cl^−^ ions), steel is susceptible to electrochemical corrosion, which leads to the deterioration of mechanical properties, reduction of service life of structures, and significant economic losses.^2^ Therefore, protecting steel against corrosion is an urgent requirement in industrial practice.^3^

Currently, various approaches are employed to protect steel from corrosion, including protective coatings, metallic plating (Zn, Ni, Cr), cathodic protection, development of corrosion-resistant alloy, and application of chemical inhibitors for corrosion prevention. Among these approaches, corrosion inhibitors are regarded as an efficient, versatile, and cost-effective strategy, particularly in acidic environments that are commonly used for pickling and cleaning metal surfaces.^4^

The demand for eco-friendly corrosion inhibitors has grown significantly in recent years, aiming to replace toxic conventional inorganic and organic inhibitors.^5^ The eco-friendly corrosion inhibitors are typically derived from natural sources or possess low toxicity, high biodegradability, and environmental compatibility.^6^ Various classes of green inhibitors have been investigated, including plant extracts, alkaloids, flavonoids, amino acids, proteins, polysaccharides, and organic species incorporating heteroatoms such as nitrogen, oxygen, and sulfur that can adsorb onto metal surfaces.^7–11^

Among these substances, vitamins constitute a promising group of bio-derived compounds that have gained significant attention as eco-friendly corrosion inhibitors.^12,13^ Owing to the presence of multiple heteroatoms and polar functional groups (–OH, –NH_2_, –COOH, heterocyclic rings) alongside conjugated π-electron systems, vitamins can interact with and adsorb onto steel surfaces through physical and/or chemical adsorption mechanisms, leading to the development of a protective coating that suppresses anodic and/or cathodic reactions.^14^ Studies have reported the use of several vitamins and their derivatives to protect steel from corrosive agents in acidic environments, including niacin (vitamin B3), pyridoxine (vitamin B6), ascorbic acid (vitamin C) and thiamine (vitamin B1).^15,16^ Research results have shown that many vitamins exhibit high inhibition efficiency, consistent with the trend toward the development of safe and sustainable metal protection materials.

Among the investigated vitamins, thiamine (vitamin B1) has considered as a potential candidate for green corrosion inhibition due to its distinctive molecular structure and strong interaction with metal surfaces.^17^ Thiamine is a heterocyclic compound consisting of a thiazolium ring containing sulfur and nitrogen linked to a pyrimidine ring bearing an amino group *via* a CH_2_ bridge, forming an electron-rich system with multiple active sites capable of electron donation and acceptance. The presence of heteroatoms such as nitrogen and sulfur, together with polar functional groups, enables thiamine molecules to adsorb onto metal surfaces through electrostatic interactions, coordination bonding, or chemical bonding with the vacant d orbitals of the metal. As a result, a protective film can be formed, reducing the rate of anodic dissolution and/or cathodic reduction reactions in acidic environments. Several recent studies have demonstrated that thiamine exhibits effective corrosion inhibition for a variety of metals and alloys. The study by M. Abdallah *et al.* on the inhibition ability of thiamine (expired vitamin) for SABIC steel in 0.5 M sulfuric acid solution reported a maximum inhibition performance of 91.14% at a concentration of 250 ppm.^18^ Electrochemical measurements confirmed that thiamine acts as a mixed-type inhibitor, controlling the kinetics of both anodic and cathodic processes, with the thiazolium ring serving as the primary adsorption center. Extending the investigation to chloride-containing corrosive environments, Y. Wen *et al.* examined thiamine in simulated concrete pore solution.^19^ The results showed that at an inhibitor concentration of 0.005 M, the protection efficiency reached 91.96%, with thiamine acting as an anodic inhibitor in NaCl medium. In addition, M. Tigori *et al.* used thiamine hydrochloride as a corrosion inhibitor for copper in 1.0 M HNO_3_.^20^ The experimental results indicated that the adsorption process on the copper surface is endothermic and that the interaction between the inhibitor and the copper surface involves both physical and chemical adsorption depending on temperature. In another study using 2.5 M HNO_3_ solution, O. K. Abiola *et al.* observed that the corrosion inhibition performance of thiamine hydrochloride for copper decreased with increasing temperature, suggesting that physical adsorption predominates under those experimental conditions.^21^ T. Chen *et al.* investigated the application of expired vitamin B1 to protect AA5083 aluminum alloy in HCl solution.^22^ The highest inhibition performance of 87.85% was observed for thiamine at 288 K. Evidence from scanning electron microscopy, atomic force microscopy and X-ray photoelectron spectroscopy analyses indicated that vitamin B1 effectively inhibits corrosion by forming a surface-adsorbed protective layer. Overall, these studies demonstrate that thiamine is a promising green corrosion inhibitor with high efficiency (>87%) for various metallic substrates.

However, most previous studies have focused on commercial thiamine or thiamine recovered from expired pharmaceutical products. Systematic investigations comparing the inhibition performance of different thiamine salts under identical experimental conditions remain limited. Therefore, further research on these salts is necessary to clarify the influence of molecular structure on inhibition efficiency and to contribute to the development of new organic corrosion inhibitor systems with enhanced performance.

**Fig. 1:**
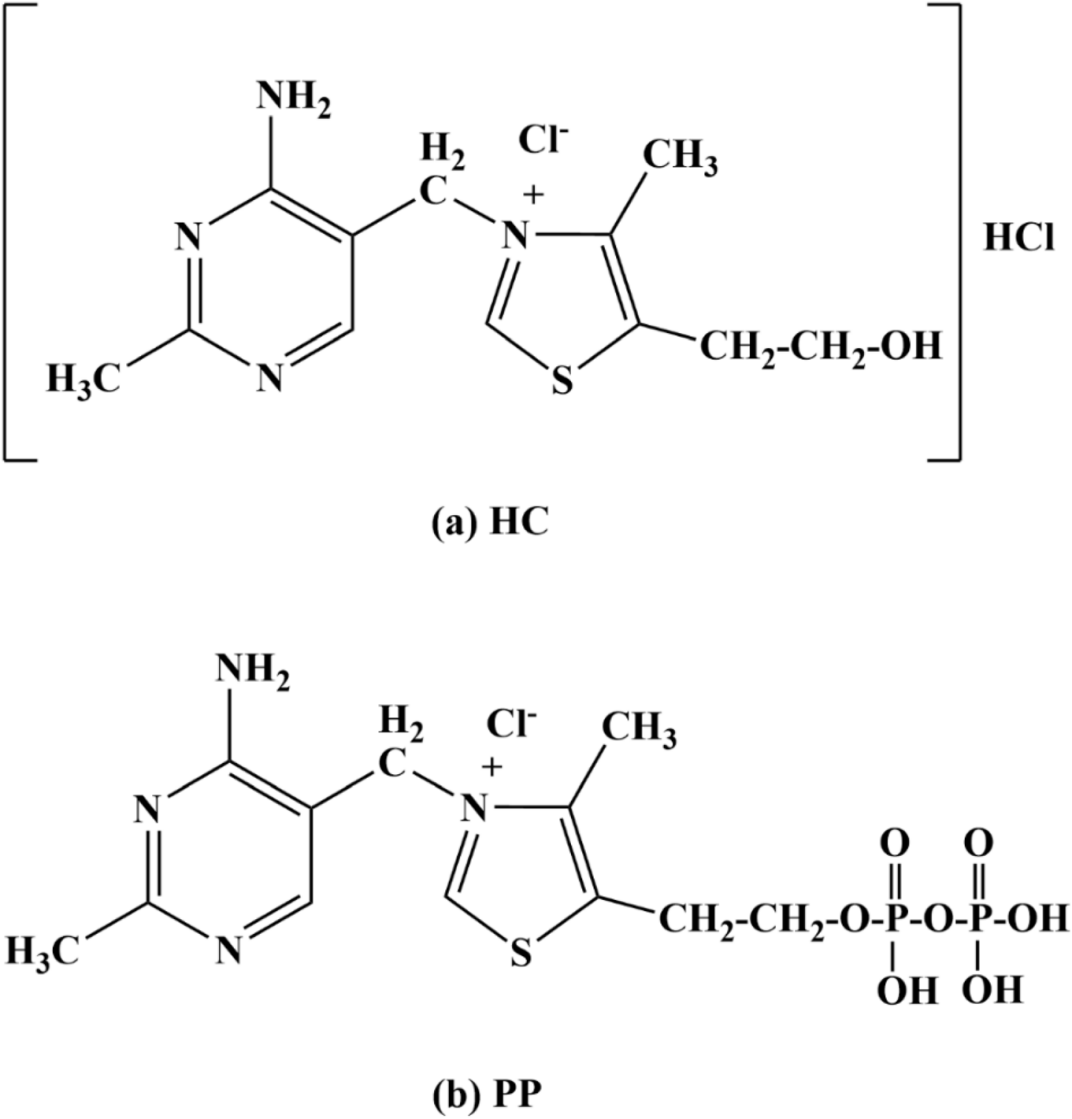
Molecular structures of (a) HC and (b) PP.

Two thiamine salts, thiamine hydrochloride (HC) and thiamine pyrophosphate (PP) (Fig. 1), are selected as promising candidates for green corrosion inhibitor systems based on both their physicochemical properties and their structural relationship. Both compounds possess heteroatom-rich frameworks (N, S) and favorable electronic characteristics that facilitate donor–acceptor interactions with the metal surface. In addition, their high water solubility and environmentally benign nature make them particularly suitable for application as green corrosion inhibitors.

From a structural perspective, HC and PP are closely related, as both are derived from thiamine and share the same thiazolium–aminopyrimidine backbone. PP represents the biologically active, phosphorylated form of HC, differing by the introduction of a pyrophosphate group. This parent–derivative relationship provides a controlled molecular platform in which the core structure is preserved while a single, chemically meaningful modification is introduced. Such a design enables a systematic evaluation of the influence of pyrophosphate functionalization, including increased charge density and the presence of additional oxygen donor sites, on adsorption behavior and corrosion inhibition performance. Moreover, the distinct functional groups in the two compounds—protonated thiazolium in HC and the oxygen-rich pyrophosphate in PP—lead to different interaction modes with the metal surface, allowing a direct comparison of adsorption mechanisms. Compared with studies employing structurally unrelated inhibitors, this approach allows the effect of functional group modification to be isolated more clearly, thereby providing deeper insight into structure–activity relationships.

The novelty of this work lies in the rational selection of a structurally related inhibitor pair to systematically elucidate corrosion inhibition mechanisms. By retaining the same heterocyclic framework and introducing only functional group modification, this study enables a assessment of how P- and O-containing groups, molecular size, and charge density govern adsorption behavior and inhibition efficiency.

In this study, the corrosion inhibition ability of the salts thiamine hydrochloride and thiamine pyrophosphate for steel in 1.0 M HCl solution will be examined through the use of both electrochemical methods and advanced computational simulations. Specifically, potentiodynamic polarization measurements will be employed to determine the corrosion current density and corrosion potential, as well as to clarify the inhibition behavior of the investigated compounds (anodic, cathodic, or mixed-type).^23^ Using electrochemical impedance spectroscopy (EIS), key electrochemical parameters such as charge transfer resistance and double-layer capacitance, as well as the features of the protective film formed on the steel surface, can be analyzed.^24^ The inhibition efficiency will subsequently be determined based on these electrochemical results.

In addition, the surface morphology and the extent of surface damage of the steel samples before and after treatment with the inhibitors will be examined using scanning electron microscopy (SEM) and energy dispersive X-ray analysis (EDX) technique in order to visually evaluate the protective capability of the inhibitors on the metal surface.^25^ Furthermore, to elucidate the protection mechanism at the molecular level, quantum chemical calculations based on density functional theory will be performed to determine important structural–electronic parameters.^26^ These parameters allow prediction of the electron-donating/accepting ability of the molecules and their tendency to adsorb onto the iron surface.^27^

Moreover, molecular dynamics (MD) simulations will be carried out to model the adsorption process of the inhibitor molecules on the Fe surface in an acidic environment, thereby determining the binding energy, optimal adsorption configuration, and molecular orientation on the metal surface.^28^ The combination of electrochemical experiments and computational simulations is expected to provide a comprehensive understanding of the corrosion inhibition mechanism and the structure–activity relationship of the investigated compounds.

## 2 Materials and methods

### 2.1 Materials

SD295B steel, with the following composition: 99.1% iron (Fe), 0.27% carbon (C), 0.55% silicon (Si), 0.04% sulfur (S), and 0.04% phosphorus (P), was used as the working electrode in this study. The steel samples were cut into cylindrical specimens and embedded in epoxy resin, leaving only a flat surface with an exposed area of 1.13 cm^2^. Mechanical polishing of the electrode surface was performed prior to each experiment using silicon carbide sandpapers (600–2000 grit), followed by rinsing with distilled water and ethanol and air drying.

Two investigated compounds, thiamine hydrochloride (C_12_H_18_Cl_2_N_4_OS; CAS number: 67-03-8; purity ≥98%) and thiamine pyrophosphate (C_12_H_19_ClN_4_O_7_P_2_S; CAS number: 154-87-0; purity >95%), were obtained from Sigma-Aldrich. Hydrochloric acid (HCl; CAS number: 7647-01-0; purity 37%) solution was prepared from analytical-grade chemicals supplied by Merck.

The blank medium was obtained by diluting HCl in double-distilled water to yield a concentration of 1.0 M. The inhibitors, HC and PP, were introduced into the blank medium to prepare solutions with concentrations of 10^−4^, 5 × 10^−4^, 10^−3^, and 5 × 10^−3^ M.

### 2.2 Methods

#### 2.2.1 Electrochemical impedance spectroscopy (EIS) measurements

EIS measurements were conducted to gain insight into the corrosion properties of steel and to examine the interaction between inhibitor molecules and the metal surface in a 1.0 M HCl.^29^ A standard three-electrode configuration was utilized for all electrochemical tests. In this setup, an SD295B steel specimen served as the working electrode, which was prepared according to the procedure described in the preceding section. In addition, a platinum electrode functioned as the counter electrode, and Ag/AgCl electrode acted as the reference electrode in this electrochemical system.

Before the impedance measurements, the working electrode was kept in the electrolyte for about 30 min to allow the electrode surface to stabilize and to attain a steady open-circuit potential (OCP) at room temperature.^30^ Subsequently, EIS measurements were carried out at the stabilized OCP by applying a small sinusoidal AC perturbation. The impedance response of the system was evaluated within a frequency domain ranging from 10^−2^ to 10^5^ Hz in order to analyze the interfacial electrochemical processes and evaluate the inhibition performance.

The experimental data displayed as Nyquist and Bode plots.^31^ From the obtained Nyquist plots, an appropriate equivalent circuit model was selected and the data were fitted using a nonlinear least-squares method in order to accurately determine the characteristic electrochemical parameters, including the solution resistance (*R*_s_), charge transfer resistance (*R*_ct_), and the constant phase element (CPE) describing the non-ideal capacitive behavior associated with the surface heterogeneity of the electrode. Each EIS measurement was performed in triplicate to ensure the reliability and reproducibility of the results. Details on the calculation of the EIS parameters are given in Table S1 of the SI.

#### 2.2.2 Potentiodynamic polarization measurements

Potentiodynamic polarization (PM) measurements were conducted using a three-electrode electrochemical cell similar to that employed for the EIS experiments. Prior to each measurement, the working electrode was placed in the test solution for about 30 min to allow the open-circuit potential to stabilize. Once a steady OCP had been established, the polarization curves were obtained by scanning the potential from −0.6 V to 0.15 V *versus* the reference electrode at 0.2 mV s^−1^ was repeated three times to ensure consistent and reliable results. The obtained polarization curves were presented as Tafel plots. From these plots, the PM parameters including the corrosion current density, the corrosion potential, the anodic and cathodic Tafel slopes were determined to evaluate the corrosion behavior of steel along with the inhibitory performance of the compounds under study.^32^

Furthermore, the impact of temperature on the polarization behavior was investigated. The experiments were conducted at temperatures between 298 and 323 K using a thermostatically controlled water bath. Before each measurement, the test solution was maintained in the water bath for 30 min to reach the desired temperature. The working electrode underwent an additional 30 min immersion before recording the polarization curves following the procedure described above.

#### 2.2.3 Surface morphology analysis

The surface characteristics of the steel after exposure to the corrosive environment were investigated using scanning electron microscopy (SEM) and energy dispersive X-ray analysis (EDX) to assess the degree of surface deterioration and the protective performance of the corrosion inhibitors.^33^ The SD295B steel specimens were prepared following the procedure described earlier and subsequently immersed in 1.0 M hydrochloric acid solution, both with and without the addition of inhibitors, for 48 h at room temperature. For the inhibited system, the inhibitor concentration used was 5 × 10^−3^ M. Upon completion of the immersion period, the specimens were taken out of the solution and gently washed with distilled water to clear any remaining solution, followed by washing with ethanol and drying in air prior to analysis. SEM images were recorded using a JSM-6010PLUS/LV microscope (JEOL). The micrographs were obtained at a magnification of 5000× to observe in detail the changes in microstructure, as well as the appearance of pits, cracks, or corrosion products on the steel surface. EDX analysis was carried out using a JSM-IT210 instrument.

#### 2.2.4 Quantum chemical calculations and molecular dynamics simulation

To gain insight into the corrosion inhibition mechanism at the molecular level, theoretical calculations were carried out using the Density Functional Theory (DFT) approach.^34^ The molecular geometries of the inhibitors were fully optimized in a simulated solvent environment in order to determine the most stable molecular structures of the inhibitor molecules (lowest energy configuration). From the optimized structures, several important electronic parameters were determined, including the energy of the highest occupied molecular orbital (*E*_HOMO_), the energy of the lowest unoccupied molecular orbital (*E*_LUMO_), the energy gap (Δ*E*_L–H_), global hardness (*η*), and molecular softness (*S*).^35^ These quantities were calculated according to the following equations:^36^

1 Δ*E*_L–H_ = *E*_LUMO_ − *E*_HOMO_

2
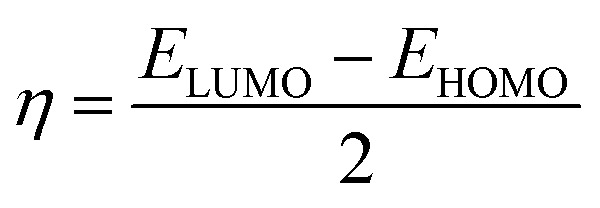
3
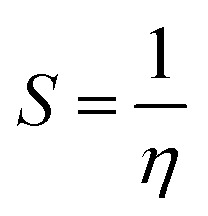


These parameters were used to evaluate the electron-donating and electron-accepting abilities of the inhibitor molecules toward the metal surface and to estimate their tendency for chemisorption *via* interactions between the frontier molecular orbitals of the inhibitors and the Fe d orbitals.^37^

In addition, molecular dynamics (MD) simulations were carried out to model the adsorption process of inhibitor molecules on the Fe surface in an acidic environment.^38^ The Fe(110) surface was constructed as a periodic crystal slab, and the simulation system consisted of the inhibitor molecule, the metal surface, and solvent molecules. During the simulation, the molecular configurations were dynamically optimized over time to reach a thermodynamic equilibrium state. The binding energy was calculated as follows:

4 *E*_binding_ = −*E*_interaction_

Higher binding energy values are indicative of stronger adsorption and improved stability at the inhibitor–metal interface.^39^

## 3 Results and discussion

### 3.1 Open circuit potential (OCP)

**Fig. 2:**
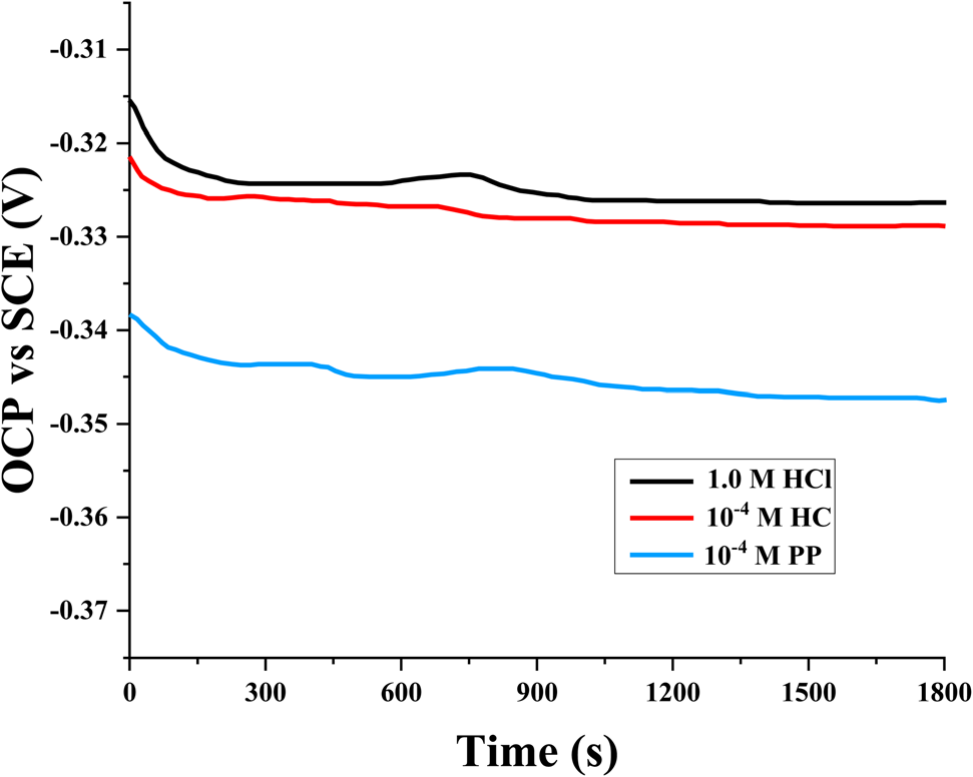
OCP of steel in the presence of HC and PP.

The dependence of OCP on immersion time for steel in 1.0 M HCl solution supplemented with 10^−4^ M HC and PP is illustrated in Fig. 2. The results show that, in both cases where inhibitors are present, the OCP values tend to shift toward more negative potentials compared with the uninhibited system. This shift reflects changes in the electrode surface state caused by the adsorption of inhibitor molecules and the restructuring of the metal/solution interfacial layer.

During the first 20 min, the OCP decreases rapidly with time. The observed phenomenon can be ascribed to the dissolution of the native oxide/hydroxide film that develops on the steel surface during air exposure.^40^ In a strongly acidic medium such as 1.0 M HCl, this passive film is rapidly destroyed, causing the metal surface to transition to an activated state.

Subsequent to an immersion period of 30 min, the OCP values reach a nearly stable state, indicating that the electrode system has established a dynamic equilibrium between metal dissolution and the adsorption/protective film formation processes of the inhibitors. Therefore, a 30 min pre-immersion time was selected to ensure surface stabilization before performing the potentiodynamic polarization measurements.

### 3.2 Electrochemical impedance spectroscopy (EIS) measurements

**Fig. 3:**
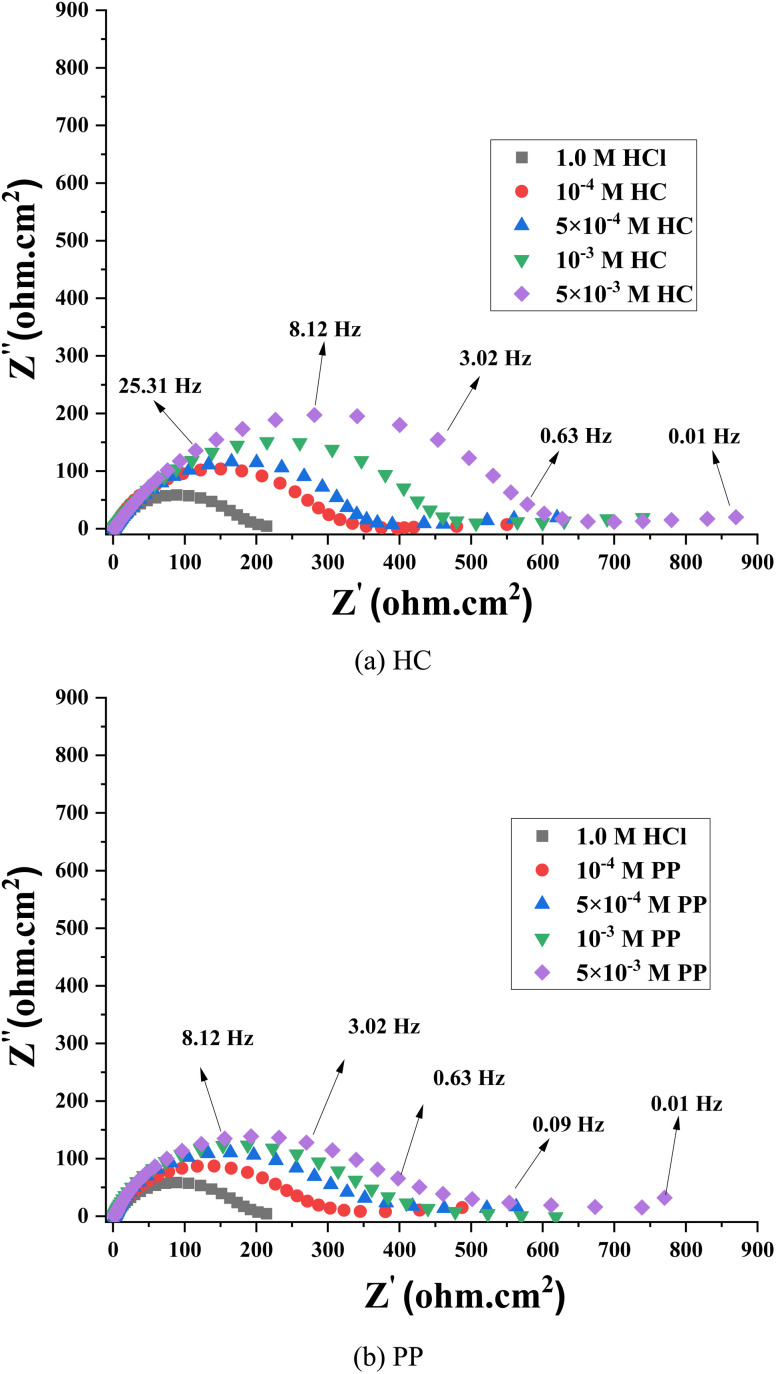
Nyquist spectra of steel in solutions containing (a) HC and (b) PP.

EIS was employed to elucidate the kinetics of the corrosion process and the adsorption mechanism of the inhibitors HC and PP on the steel surface in 1.0 M HCl solution.^41^ The Nyquist plots of steel in the investigated medium in the presence of various concentrations of HC and PP are presented in Fig. 3. All spectra exhibit depressed semicircles, which are characteristic of a non-ideal electrochemical system due to the microscopic heterogeneity of the electrode surface. The radius of the semicircle increases progressively with increasing concentrations of HC and PP, indicating an increase in the charge transfer resistance (*R*_ct_). This behavior implies that the anodic dissolution rate of steel is reduced due to the presence of a protective adsorbed layer.^42^

**Fig. 4:**
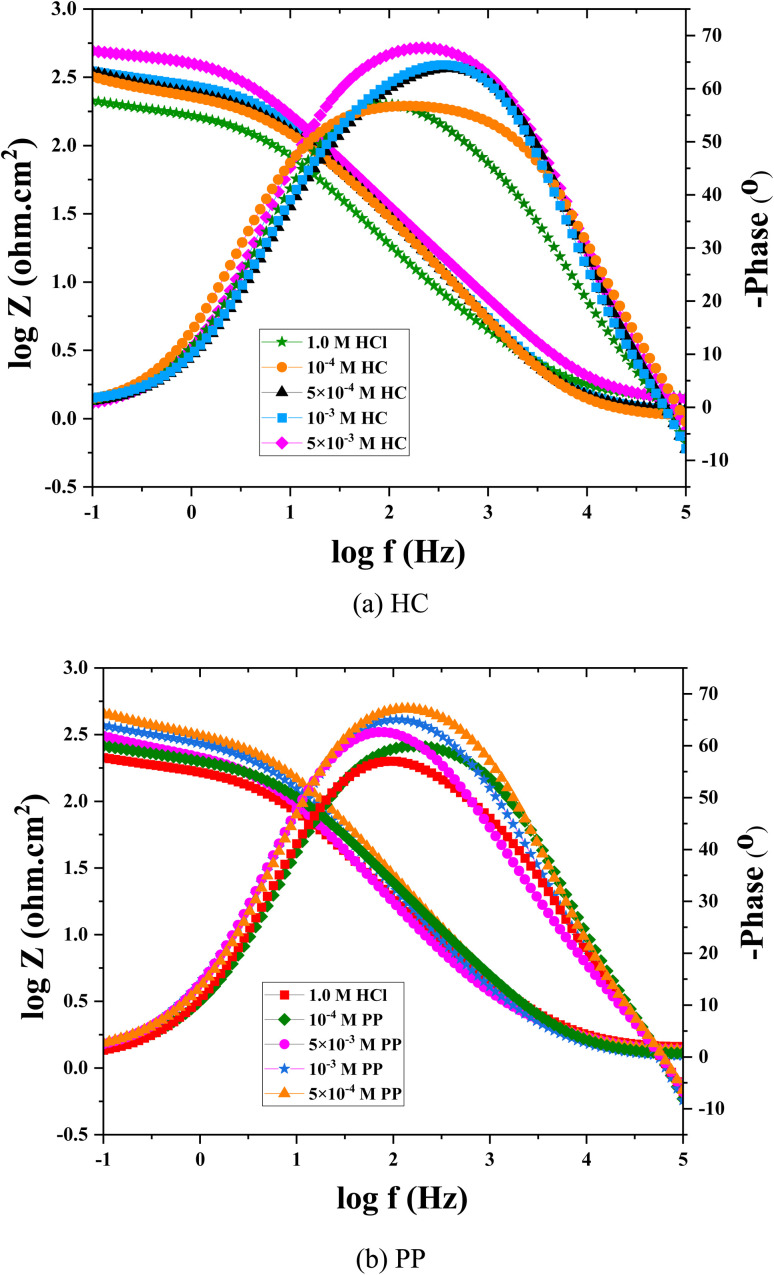
Bode magnitude and phase angle curves obtained for steel in uninhibited and inhibited solutions: (a) HC and (b) PP.

The Bode magnitude and Bode phase plots (Fig. 4) provide further information on the electrochemical characteristics of the system.^42^ As the inhibitor concentration increases, the maximum phase angle value increases and the frequency range with high phase angles becomes broader, reflecting an improvement in the dielectric properties of the protective film. However, the maximum phase angle in all cases remains lower than 90°, indicating the non-ideal capacitive behavior of the double layer,^43^ which is commonly represented by a constant phase element (CPE) instead of an ideal capacitor. Meanwhile, the log|*Z*| values in the low-frequency region increase significantly in the presence of the inhibitors, indicating an increase in the overall impedance of the system due to the enhanced surface coverage and adsorption of HC and PP molecules on the steel surface.

Fig. S1 illustrates the agreement between the experimental data and the simulated Nyquist plots for steel in 1.0 M HCl with HC and PP at 10^−4^ M. The simulated curves obtained using Thales 4.5 software show a high degree of agreement with the experimental data, as confirmed by the low chi-square (*χ*^2^ = 0.001–0.006)) values given in Table 1. This result demonstrates that the selected equivalent circuit model is appropriate for describing the investigated electrochemical system.

**Table 1:** Simulation results obtained from the EIS measurements

Inhibitors	*C*_(inh)_ (M)	*R*_s_ (Ω cm^2^)	*R*_1_ (Ω cm^2^)	CPE_1_ (µF cm^−2^)	*n*_1_	*R*_2_ (Ω cm^2^)	CPE_2_ (µF cm^−2^)	*n*_2_	*R*_p_ (Ω cm^2^)	% *η*_EIS_	*χ* ^2^
Blank		1.482	183.5	44.25	0.91	16.78	76.85	0.86	200.28 ± 3.35		0.003
HC	10^−4^	1.155	297.9	32.1	0.82	790.65	67.26	0.75	1088.55 ± 4.09	81.60 ± 0.32	0.002
	5 × 10^−4^	1.321	349.3	26.73	0.73	898.31	64.93	0.83	1247.61 ± 5.17	83.95 ± 0.28	0.001
	10^−3^	1.069	437.9	12.97	0.80	968.94	61.54	0.75	1406.84 ± 7.53	85.76 ± 0.25	0.001
	5 × 10^−3^	1.166	556.5	9.02	0.79	1094.79	60.89	0.84	1651.29 ± 12.54	87.87 ± 0.22	0.002
PP	10^−4^	1.288	246.6	38.03	0.77	697.95	111.9	0.80	944.55 ± 8.10	78.80 ± 0.40	0.004
	5 × 10^−4^	1.459	323.4	35.58	0.76	763.19	100.7	0.69	1086.59 ± 21.39	81.57 ± 0.48	0.006
	10^−3^	1.275	361.8	27.42	0.85	887.94	98.3	0.71	1249.74 ± 65.67	83.97 ± 0.88	0.001
	5 × 10^−3^	1.367	364.2	20.67	0.69	996.52	92.2	0.74	1360.72 ± 52.13	85.28 ± 0.62	0.002

**Fig. 5:**
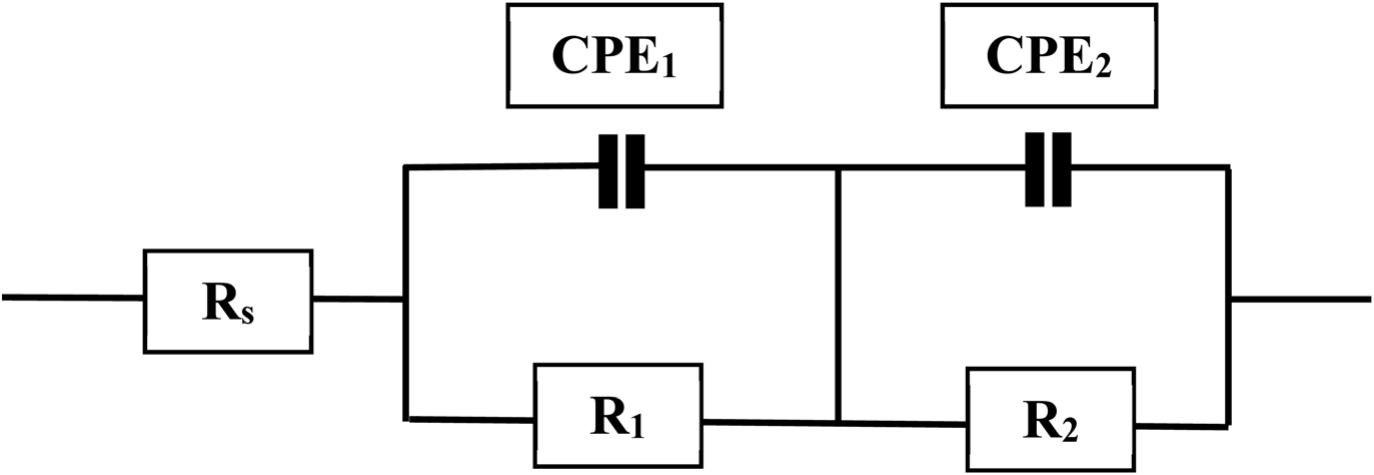
Simulated impedance plots of steel in the presence of HC and PP.

The Nyquist plots were analyzed using an equivalent circuit model consisting of the solution resistance (*R*_s_) in series with two parallel branches *R*_1_(CPE_1_) and *R*_2_(CPE_2_), as shown in Fig. 5. In this model, *R*_1_ and *R*_2_ correspond to the resistance of the adsorbed film and the charge transfer resistance at the metal/solution interface, respectively, while CPE_1_ and CPE_2_ describe the non-ideal capacitive behavior of the double layer and the protective film. This two-time-constant structure is consistent with a two-layer surface film model, consisting of the inhibitor adsorption layer and the electrical double layer at the steel/solution interface.^44^

The calculated EIS parameters and inhibition efficiencies are listed in Table 1. The results show that with increasing concentrations of HC and PP, the values of *R*_1_ and *R*_2_ gradually increase to an optimal value, reflecting the enhancement of surface impedance and the increased resistance to the charge transfer process. In contrast, the values of CPE_1_ and CPE_2_ decrease, indicating a reduction in the effective capacitance of the double layer due to the replacement of water molecules by inhibitor molecules with lower dielectric constants.^45^ The *n* parameters of the CPE are lower than 1, indicating a deviation from ideal capacitive behavior, which originates from surface roughness, heterogeneity, the non-uniform distribution of active sites on the surface, and the partial coverage of the surface by the adsorbed protective film.

The corrosion rate can be evaluated through the polarization resistance (*R*_p_) parameter. From this value, the inhibition efficiency (*η*_EIS_%) was computed *via* the formula below:^46^5
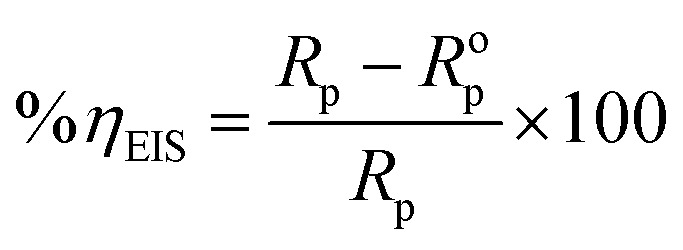
In this expression, *R*^o^_p_ and *R*_p_ denotes the polarization resistances of the blank sample without and with inhibitor.

6 *R*_p_ = *R*_1_ + *R*_2_

The inhibition efficiency (*η*_EIS_%) of HC ranges from 81.60% to 87.87% as the concentration increases from 10^−4^ M to 5 × 10^−3^ M (Table 1). For PP, the *η*_EIS_% values vary from 78.80% to 85.28% within the same concentration range. These values indicate that both HC and PP exhibit effective corrosion inhibition performance in 1.0 M HCl solution, with the protection mechanism mainly attributed to the adsorption process forming a barrier film on the steel surface, thereby reducing the charge transfer rate and limiting metal dissolution.

In comparison, HC exhibits higher corrosion inhibition efficiency than PP at all investigated concentrations. This difference may be related to variations in molecular structure, electron density on heteroatom active sites, and the stronger electron-donating/accepting ability of HC. As a result, HC can interact more strongly with the steel surface, forming a more stable protective adsorption film compared with PP.

### 3.3 Potentiodynamic polarization measurements (PM)

**Fig. 6:**
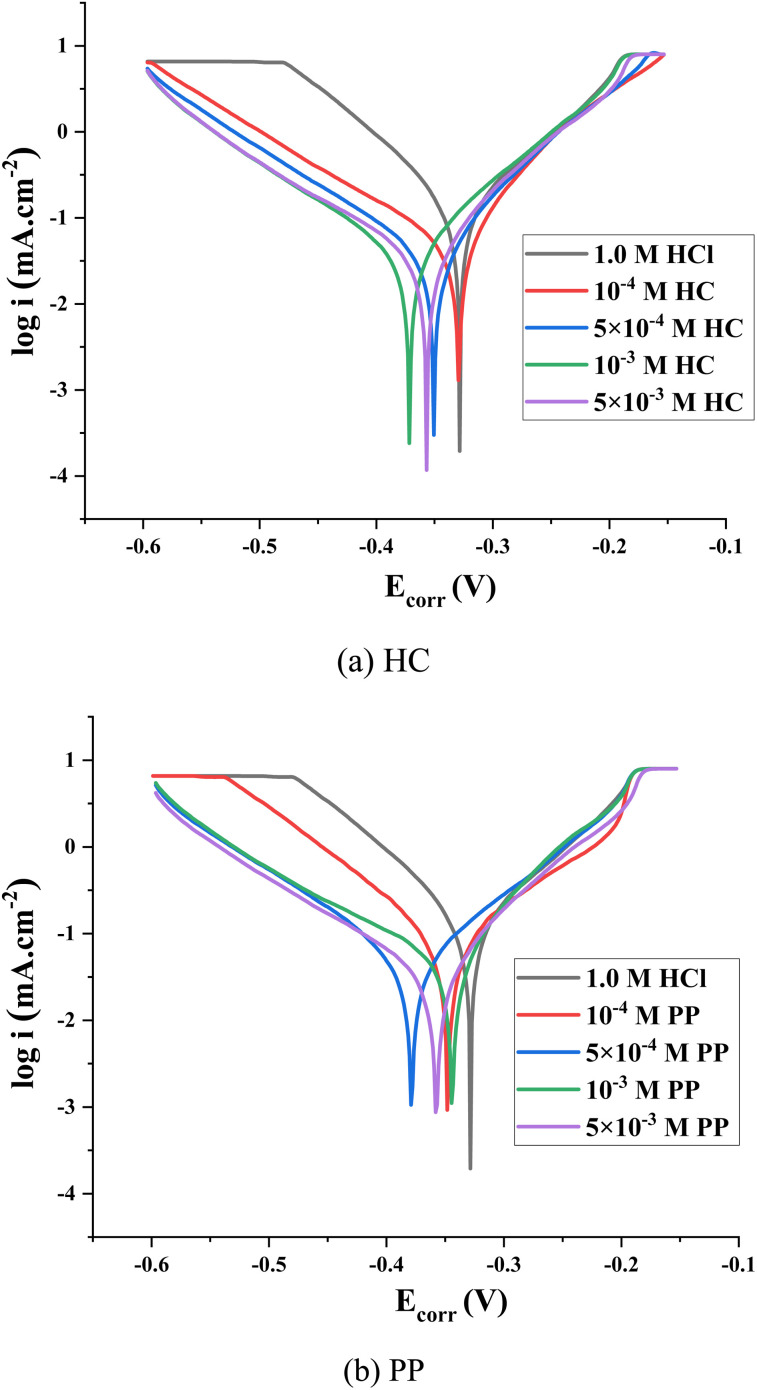
Tafel diagrams of steel in uninhibited and inhibited conditions: (a) HC and (b) PP.

Potentiodynamic polarization techniques were employed to assess the corrosion inhibition characteristics of HC and PP in 1.0 M HCl solution.^47^ The experiments were performed at inhibitor concentrations between 10^−4^ M and 5 × 10^−3^ M. The obtained polarization curves are illustrated in the form of Tafel plots, as shown in Fig. 6.

The results show that both inhibitors exhibit a similar trend: as the concentration increases, the entire polarization curves shift toward lower current densities, particularly in the negative potential direction. This shift reflects a significant decrease in the corrosion current density (*i*_corr_), indicating that increasing the inhibitor concentration leads to improved inhibition efficiency. This phenomenon can be explained by the enhanced adsorption of inhibitor molecules onto the steel surface, forming a protective film capable of simultaneously hindering both the anodic metal dissolution and the cathodic reduction reaction.

The shift in corrosion potential (*E*_corr_) in the presence of HC and PP is less than 85 mV compared with the blank solution. According to the commonly used classification criteria in Tafel analysis, when ∣*Δ*_Ecorr_∣ < 85 mV, the inhibitor is considered a mixed-type inhibitor. This indicates that HC and PP simultaneously affect both electrochemical processes: (i) the metal oxidation reaction at the anodic branch, and (ii) the proton reduction reaction at the cathodic branch.

Table 2 displays the electrochemical parameters obtained from Tafel extrapolation, namely the corrosion current density (*i*_corr_), anodic Tafel slope (*β*_a_), cathodic Tafel slope (*β*_c_), and, corrosion potential (*E*_corr_). Based on these corrosion current density values, the corrosion inhibition efficiency (*η*_PM_%) was computed *via* the fomula below:^48^7
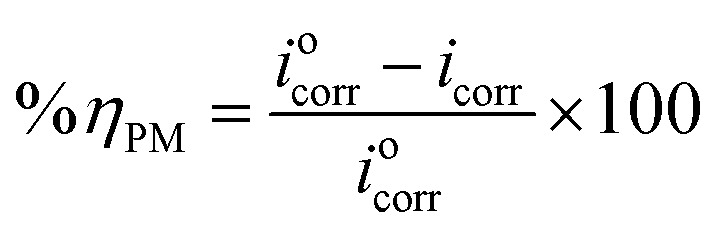
In this equation, *i*^o^_corr_ denotes the corrosion current density obtained for the uninhibited system, while *i*_corr_ refers to the corrosion current density recorded when the inhibitor is added.

**Table 2:** Results of the Tafel measurements at 298 K

Inhibitors	Concentration (M)	*β*_a_ (mV dec^−1^)	*β*_c_ (mV dec^−1^)	*E*_corr_ (mV)	*i*_corr_ (µA cm^−2^)	% *η*_PM_
Blank		0.087	−0.087	−0.325	124.66 ± 2.07	
HC	10^−4^	0.078	−0.131	−0.328	16.05 ± 0.15	87.12 ± 0.25
	5 × 10^−4^	0.064	−0.120	−0.346	13.08 ± 0.12	89.51 ± 0.20
	10^−3^	0.056	−0.118	−0.370	12.30 ± 0.09	90.14 ± 0.18
	5 × 10^−3^	0.048	−0.108	−0.355	10.75 ± 0.07	91.38 ± 0.15
PP	10^−4^	0.067	−0.126	−0.345	21.60 ± 0.31	82.67 ± 0.38
	5 × 10^−4^	0.053	−0.117	−0.375	19.52 ± 0.27	84.34 ± 0.34
	10^−3^	0.049	−0.114	−0.343	16.62 ± 0.30	86.67 ± 0.33
	5 × 10^−3^	0.042	−0.101	−0.360	14.48 ± 0.25	88.38 ± 0.28

The Tafel slopes obtained from potentiodynamic polarization measurements enable a quantitative evaluation of the influence of inhibitors on the kinetics of electrochemical reactions occurring on the steel surface in 1.0 M HCl solution. For the uninhibited HCl solution, the anodic Tafel slope (*β*_a_) is 0.087 V dec^−1^, while the cathodic Tafel slope (*β*_c_) is −0.087 V dec^−1^. These values are characteristic of the iron dissolution process at the anodic branch and the proton reduction reaction at the cathodic branch in an acidic medium.

Upon the addition of HC, the *β*_a_ values decrease and fall within the range of 0.048–0.078 V dec^−1^, while *β*_c_ varies from −0.131 to −0.108 V dec^−1^. Similarly, for PP, the *β*_a_ values lie in the range of 0.042–0.067 V dec^−1^, whereas *β*_c_ ranges from −0.126 to −0.101 V dec^−1^. The observation that |*β*_c_| values are greater than *β*_a_ in both the HC and PP inhibitors indicates that the cathodic process is more strongly affected than the anodic one. These results confirm that HC and PP acts as a mixed-type inhibitor, with a predominant influence on the cathodic reaction.

Table 2 shows that the inhibition efficiencies of HC reach 87.12%, 89.51%, 90.14%, and 91.38% corresponding to concentrations from 10^−4^ M to 5 × 10^−3^ M. Meanwhile, PP exhibits inhibition efficiencies of 82.67%, 84.34%, 86.67%, and 88.38% under the same conditions at 298 K. A quantitative comparison indicates that at all investigated concentrations, HC consistently exhibits lower *i*_corr_ values and higher inhibition efficiencies than PP. This suggests that HC has a stronger adsorption capability and generates a more stable and protective layer on the steel surface, thereby demonstrating superior corrosion inhibition performance compared with PP in the investigated acidic environment.

### 3.4 Effect of temperature on corrosion efficiency

The temperature dependence of the inhibitory efficiency of HC and PP toward steel in 1.0 M HCl solution was analyzed in the range of 298–323 K to gain insight into the nature of the inhibitor–metal surface interaction. The inhibition efficiency values (*η*_PM_%) obtained from polarization measurements are summarized in Tables 2 and 3. In Table 3, the values listed in parentheses correspond to the mean absolute deviation for corrosion inhibition efficiency measurements.

**Table 3:** Evaluation of the corrosion inhibition performance of HC and PP for steel under various temperature conditions

Inhibitors	Concentration (M)	303 K	313 K	323 K
HC	10^−4^	82.58 (0.74)	74.46 (0.73)	69.70 (1.03)
	5 × 10^−4^	85.81 (1.05)	77.30 (1.04)	71.27 (0.94)
	10^−3^	86.65 (0.41)	79.00 (0.55)	74.24 (1.12)
	5 × 10^−3^	88.32 (0.48)	82.41 (0.46)	80.00 (0.61)
PP	10^−4^	78.06 (0.54)	68.17 (0.85)	65.53 (0.63)
	5 × 10^−4^	80.15 (0.84)	71.18 (0.52)	68.17 (0.75)
	10^−3^	82.12 (0.75)	74.34 (0.48)	72.65 (0.82)
	5 × 10^−3^	84.95 (0.88)	79.05 (0.76)	77.56 (0.95)

For HC, at 298 K the inhibition efficiency ranged from 87.12% to 91.38% as the concentration increased from 10^−4^ M to 5 × 10^−3^ M. When the temperature increased to 303 K, the efficiency decreased to 82.58–88.32%. At 313 K, this value further decreased to 74.46–82.41%, and at 323 K it dropped to 69.70–80.00%. The decreasing trend in efficiency with increasing temperature indicates that the protective ability of HC gradually diminishes as the environment becomes more aggressive.

Similarly, for PP, the inhibition efficiency reached 82.67–88.38% at 298 K; decreased to 78.06–84.95% at 303 K; further declined to 68.17–79.05% at 313 K; and reached the lowest values of 65.53–77.56% at 323 K. Although PP still exhibits inhibition performance throughout the investigated temperature range, its efficiency is consistently lower than that of HC under the same concentration and temperature conditions.

A common feature observed for both inhibitors is that, at the same concentration, the inhibition efficiency at lower temperatures is always higher than that at elevated temperatures. This phenomenon can be explained by the increase in the corrosion reaction rate according to the Arrhenius equation as temperature rises,^49^ leading to a higher corrosion current density. At the same time, at higher temperatures the desorption of inhibitor molecules from the metal surface becomes more favorable, which reduces the surface coverage (*θ*) and weakens the protective film.

The observed decline in inhibition efficiency at elevated temperatures generally suggests that the adsorption mechanism of HC and PP on the steel surface involves a significant contribution from physical adsorption, which is sensitive to temperature. However, the fact that the inhibition efficiency remains relatively high even at 323 K indicates that chemical adsorption may also occur simultaneously, contributing to the stabilization of the protective film.

Overall, the findings imply that both HC and PP act as effective inhibitors within the investigated temperature range; however, HC exhibits superior protective performance and better thermal stability compared with PP.

### 3.5 Adsorption isotherms

To investigate the adsorption characteristics of HC and PP on the steel surface in acidic environment, two commonly used adsorption isotherm models, the Temkin and Langmuir models, were applied.^50,51^

Temkin isotherm:8
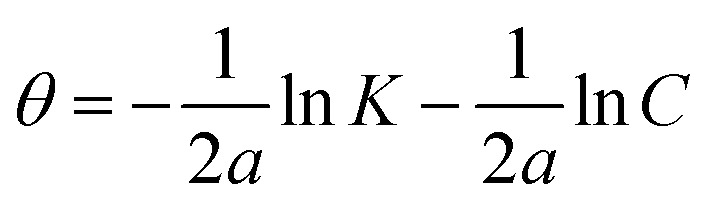
In which, *a* reprensents the lateral molecular interaction. A positive value of *a* indicates attraction, while a negative value signifies repulsion.^43^*θ* denotes the surface coverage, determined from the experimental inhibition efficiency. *K* denotes the equilibrium constant associated with the adsorption process and *C* is the inhibitor concentration.

Langmuir isotherm:9
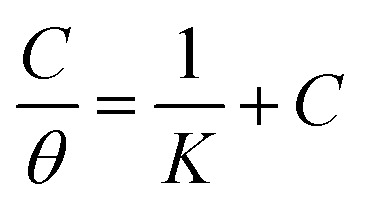


**Fig. 7:**
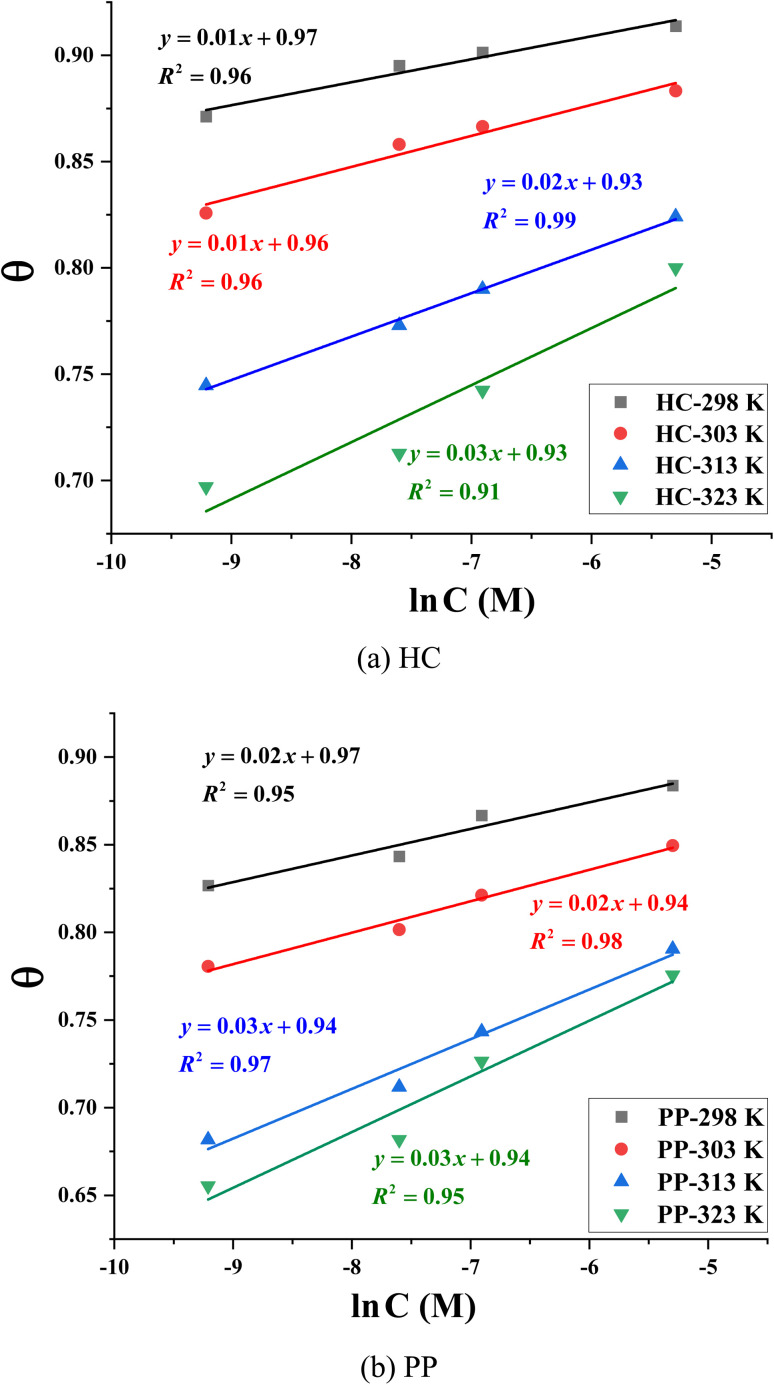
Temkin adsorption isotherm model of (a) HC and (b) PP.

The corresponding linear plots were constructed based on the relationships between ln *C* and *θ* (Temkin) and between *C*/*θ* and *C* (Langmuir). For the Temkin model (Fig. 7), the linear correlation coefficients (*R*^2^) for the plots of ln *C versus θ* range from 0.91 to 0.99 (Table 4). The relatively low *R*^2^ values—specifically 0.91 at 323 K for HC and 0.95 at 298 K and 323 K for PP—indicate noticeable deviations from linearity. These results suggest that the adsorption of HC and PP on the steel surface is not adequately described by the Temkin isotherm.

**Table 4:** Parameters results from various models of isotherms

Isotherms	Temperature (K)	Inhibitors	Parameter		*R* ^2^
Temkin	298	HC	*a*	−46.30	0.96
	303			−34.25	0.96
	313			−24.51	0.99
	323			−18.66	0.91
	298	PP		−32.89	0.95
	303			−27.93	0.98
	313			−17.61	0.97
	323			−15.72	0.95
Langmuir	298	HC	Slope	1.09	0.99
	303			1.13	0.99
	313			1.21	0.99
	323			1.23	0.99
	298	PP		1.13	0.99
	303			1.17	0.99
	313			1.25	0.99
	323			1.26	0.99

**Fig. 8:**
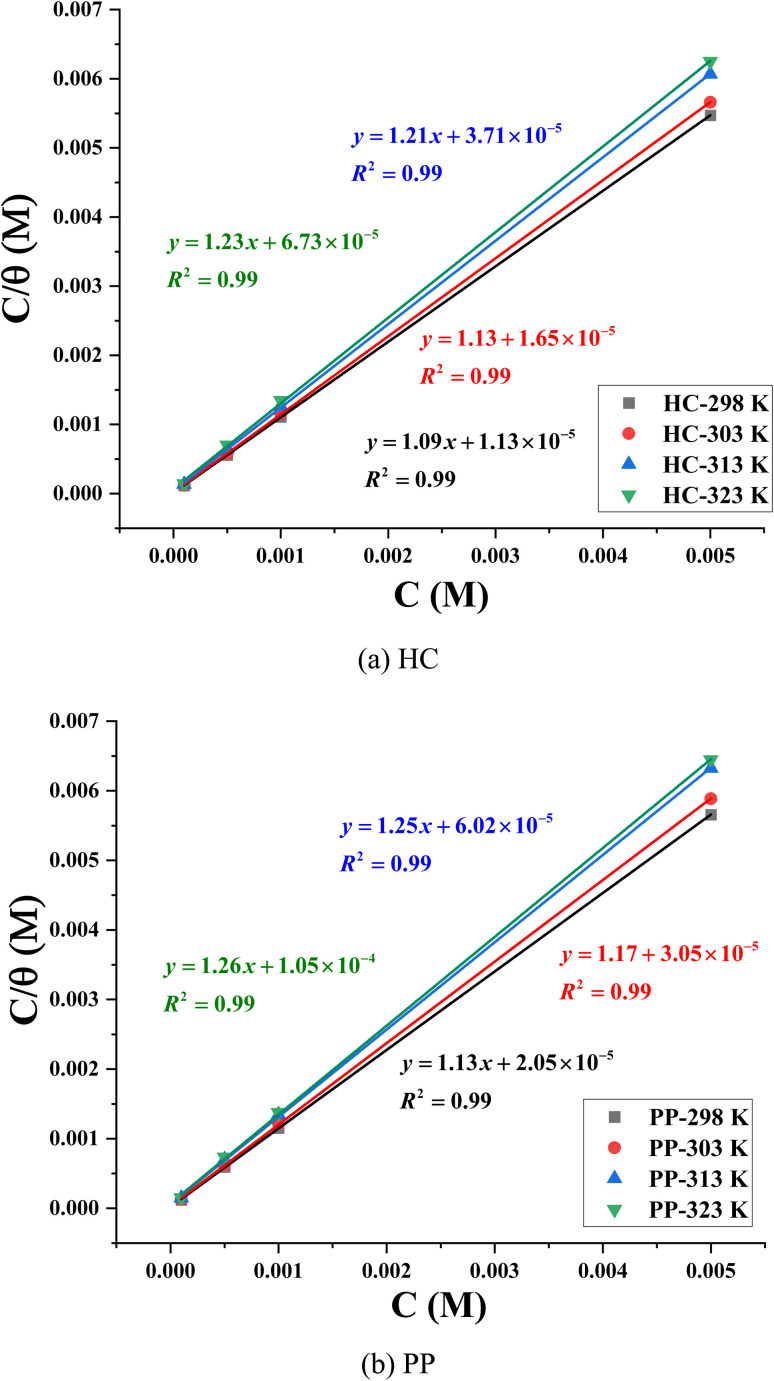
langmuir adsorption isotherm model of (a) HC and (b) PP.

In contrast, for the Langmuir model (Fig. 8), the plots of *C*/*θ versus C* exhibit excellent linearity, with correlation coefficients (*R*^2^) close to 0.99 for both HC and PP. These values indicate that the experimental data are well described by the Langmuir equation. However, the slopes of the fitted lines deviate slightly from the ideal value of unity, ranging from 1.09 to 1.23 for HC and from 1.13 to 1.26 for PP (Table 4). This deviation suggests that the adsorption process does not strictly satisfy the fundamental assumptions of the classical Langmuir model, such as a perfectly homogeneous surface and the absence of interactions between adsorbed species.^52^

### 3.6 Thermodynamic parameters of adsorption process

Based on the Langmuir isotherm model, the adsorption equilibrium constant *K* was calculated from the intercept of the linear relationship between *C*/*θ* and *C*. This constant provides information about the affinity of inhibitor molecules toward the steel surface, where larger K values correspond to stronger adsorption and a more stable protective layer.

The standard Gibbs free energy of adsorption (Δ*G*°) was calculated from the adsorption constant using the thermodynamic expression:^53^

10 Δ*G*° = −*RT* ln(55.5 K)

In this equation, 55.5 shows the molar concentration of water in the solution (mol L^−1^). After determining Δ*G*°, the standard enthalpy (Δ*H*°) and entropy (Δ*S*°) changes of adsorption were evaluated using the thermodynamic relationship:

11 Δ*G*° = Δ*H*° − *T*Δ*S*°

**Fig. 9:**
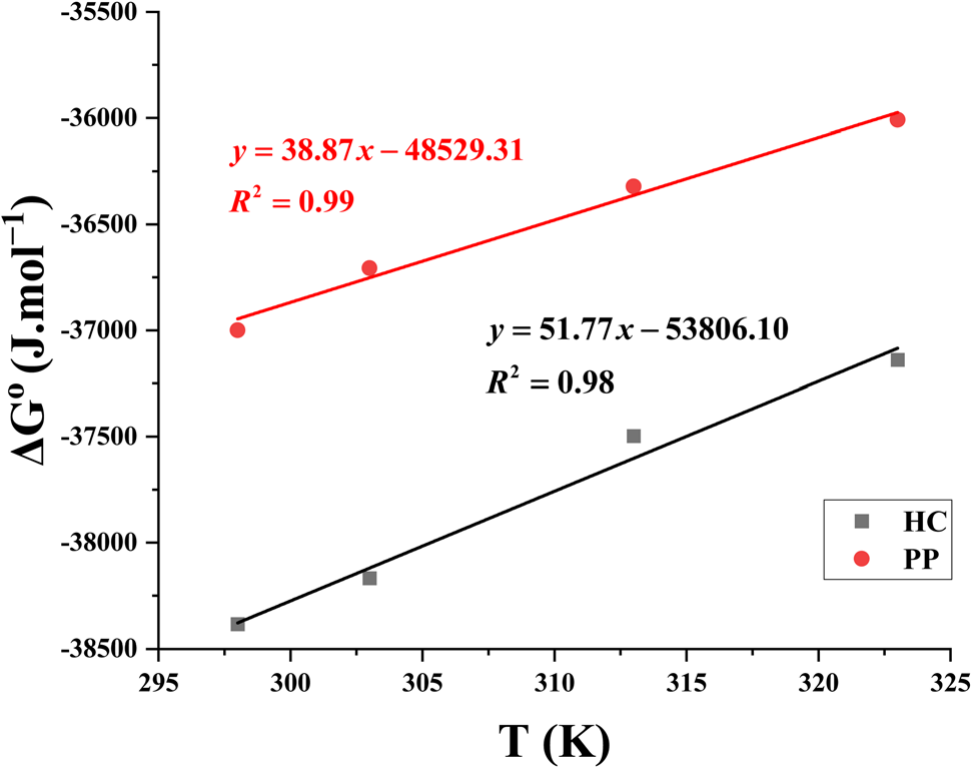
Linear plot showing the relationship between temperature and the standard Gibbs free energy of adsorption.

By rearranging this equation into a linear form, Δ*G*° was plotted as a function of temperature (*T*), as shown in Fig. 9. The resulting plots for HC and PP exhibit good linearity with high correlation coefficients, indicating the reliability of the applied approach. From these linear plots, the slope corresponds to −Δ*S*°, while the intercept represents Δ*H*°. Accordingly, the values of Δ*H*° and Δ*S*° for adsorption were determined and summarized in Table 5.

**Table 5:** Thermodynamic characteristics of HC and PP adsorption on the steel surface at different temperatures

Inhibitors	Temperature (K)	K (M^−1^)	Δ*G*^o^ (J mol^−1^)	Δ*H*^o^ (J mol^−1^)	Δ*S*^o^ (J mol^−1^ K^−1^)
HC	298	96 374.89	−38383.50	−53806.10	−51.77
	303	68 484.85	−38166.89		
	313	32 614.56	−37496.01		
	323	18 276.37	−37138.71		
PP	298	55 121.95	−36999.28	−48261.94	−37.79
	303	33 918.13	−36396.79		
	313	18 865.03	−36071.40		
	323	11 809.52	−35965.98		

The illustrated results in Table 5 indicate that *K* value decreases much as the temperature increases. Specifically, for HC, *K* decreases from 96 374.89 M^−1^ to 18 276.37 M^−1^; meanwhile, for PP, this value decreases from 55 121.95 M^−1^ to 11 809.52 M^−1^. The observed decrease in K with rising temperature implies that the adsorption process is weakened at higher temperatures. Moreover, at the same investigated temperature, the *K* values of HC are consistently higher than those of PP, demonstrating that HC has a stronger adsorption affinity and forms a more stable protective layer on the steel surface.

The negative values of Δ*G*° for both inhibitors confirm that the adsorption process occurs spontaneously. The exothermic nature of the adsorption process is determined by the negative values of Δ*H*°. Furthermore, the negative entropy change (Δ*S*° < 0) indicates a decrease in the degree of disorder at the metal/solution interface. This behavior can be attributed to the adsorption of HC or PP molecules onto the steel surface, which leads to the formation of an organized and structured adsorbed layer.

### 3.7 Surface morphology analysis

To evaluate the level of surface degradation and the protective effect of the investigated inhibitors, the morphology of the steel surface before and after immersion in 1.0 M HCl solution, with and without inhibitors, was characterized using scanning electron microscopy (SEM).^54^

**Fig. 10:**
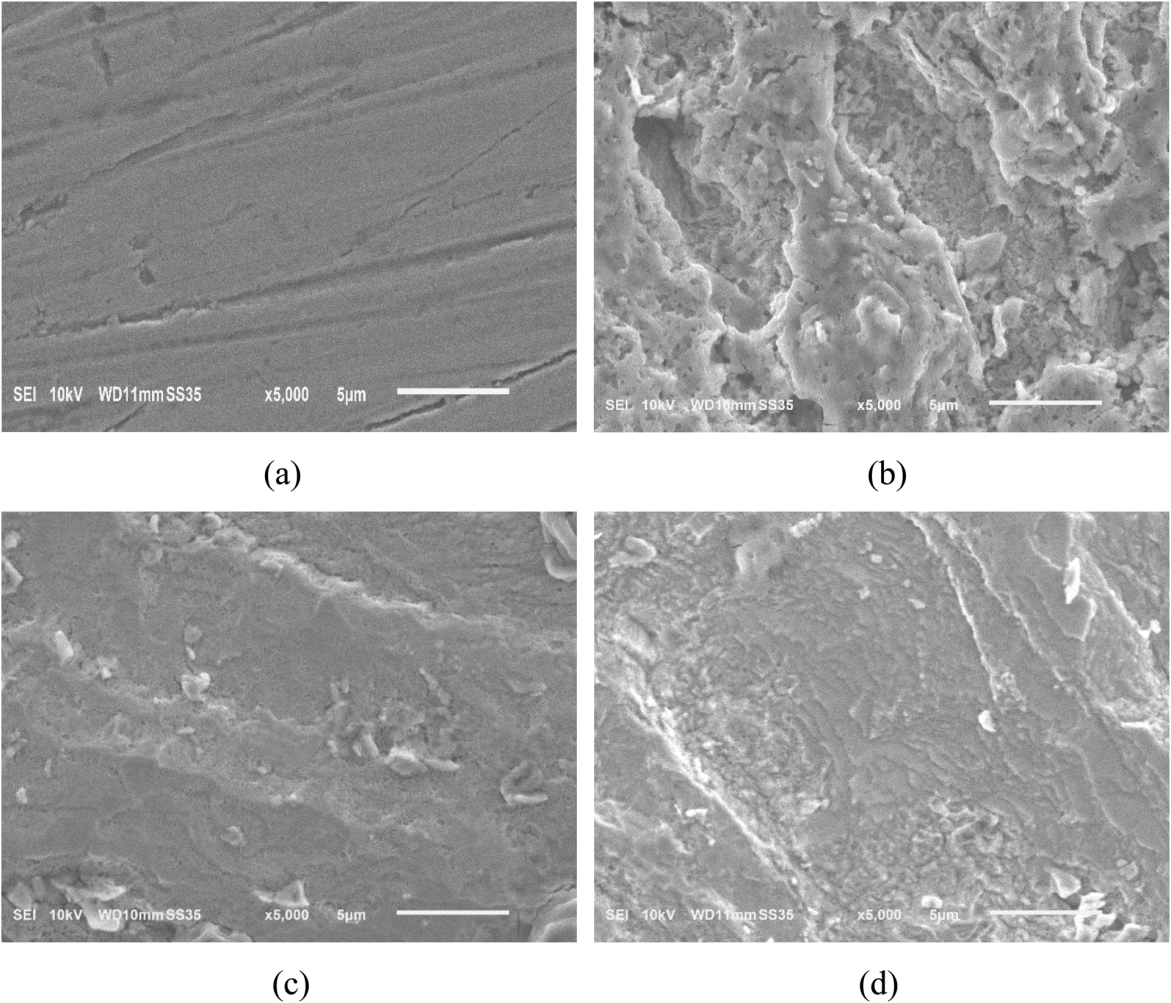
SEM images of steel surface: (a) before corrosion, (b) after corrosion, and in the presence of (c) HC and (d) PP.

Before exposure to the corrosive medium, the steel surface exhibited a relatively uniform, smooth, and continuous structure, with no observable defects such as pits, pores, or localized corrosion regions (Fig. 10a). This indicates that the initial steel sample possessed a good surface condition, which is suitable for evaluating morphological changes after corrosion. After immersion in 1.0 M HCl solution without any inhibitor, the steel surface was severely damaged (Fig. 10b). The SEM image reveals the dense presence of corrosion pits, cracks, and localized corrosion regions of various sizes. The surface structure becomes rough and heterogeneous, indicating that the metal dissolution process occurs intensely due to the aggressive acidic environment, particularly under the attack of Cl^−^ ions.^55^

In contrast, when the steel was immersed in HCl solution containing the inhibitors HC and PP, the metal surface was significantly improved (Fig. 10c and d). The SEM images show that the number and size of corrosion pits were markedly reduced, and the surface became relatively smoother and exhibited fewer defects compared with the sample without inhibitor. This observation highlights that the inhibitor molecules create a surface-bound film upon adsorption, effectively shielding the metal from corrosive attack. Comparing the two inhibitors, the steel sample in the presence of HC exhibits a smoother and more uniform surface than the sample containing PP. The defect density and the degree of surface damage in the presence of HC are lower, indicating that the protective film formed by HC is more continuous and stable. These results are consistent with the electrochemical data, confirming that HC exhibits superior corrosion inhibition performance compared with PP in 1.0 M HCl solution.

Energy-dispersive X-ray (EDX) spectroscopy was employed to characterize the surface elemental composition of steel samples immersed in 1.0 M HCl solution in the absence and presence of inhibitors.^56^ The corresponding spectra are presented in Fig. S2, while the quantitative atomic percentages are summarized in Table 6. In the uninhibited solution, a high oxygen content of 56.66% was observed, indicating the extensive formation and accumulation of corrosion products, primarily iron oxides and hydroxides, on the steel surface. Upon the addition of inhibitors, the oxygen content decreased significantly to 29.63% for HC and 47.70% for PP, demonstrating the effective suppression of corrosion processes and a consequent reduction in oxide/hydroxide layer formation. Furthermore, in the presence of PP, trace amounts of phosphorus (0.01%) and sulfur (0.19%) were detected, providing clear evidence for the adsorption of PP molecules onto the steel surface.

**Table 6:** Atomic percentage content of elements obtained from EDX spectra

%Atom	HCl	HC	PP
C	6.58	9.78	6.29
N	2.21	1.03	0.87
O	56.66	29.63	47.7
Cl	1.61	3.52	5.76
Fe	32.94	56.05	39.18
P	0.00	0.00	0.01
S	0.00	0.00	0.19

The iron content in the uninhibited system was relatively low (32.94%) due to the substantial coverage of the surface by oxygen-rich corrosion products. In contrast, the Fe content increased markedly in the inhibited systems, reaching 56.05% for HC and 39.18% for PP. This increase is attributed to the inhibition of corrosion, which limits the formation of oxygen-containing species on the surface. Concurrently, the adsorption of inhibitor molecules leads to the formation of a thin and protective film, thereby restricting the access of aggressive ions to the metal surface and resulting in a higher relative Fe signal in the EDX spectra.^43^

### 3.8 Quantum chemical calculations

**Fig. 11:**
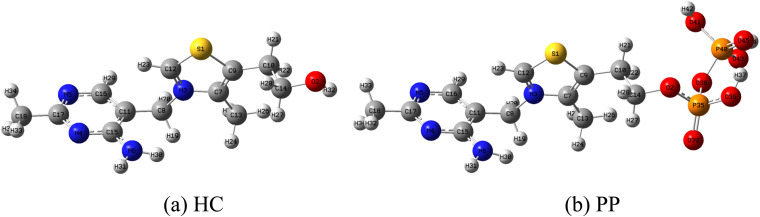
Optimized structures of (a) HC and (b) PP in water.

To provide molecular-level insight into the adsorption behavior and corrosion inhibition mechanism of HC and PP, quantum chemical calculations were employed to analyze their geometric and electronic structures.^57^ Geometry optimization was initially performed in water using DFT at the B3LYP/6-311G(d,p) level (Fig. 11). Frequency calculations were then performed to confirm that the optimized configurations represent stable energy minima, indicated by the lack of imaginary vibrational frequencies.

**Fig. 12:**
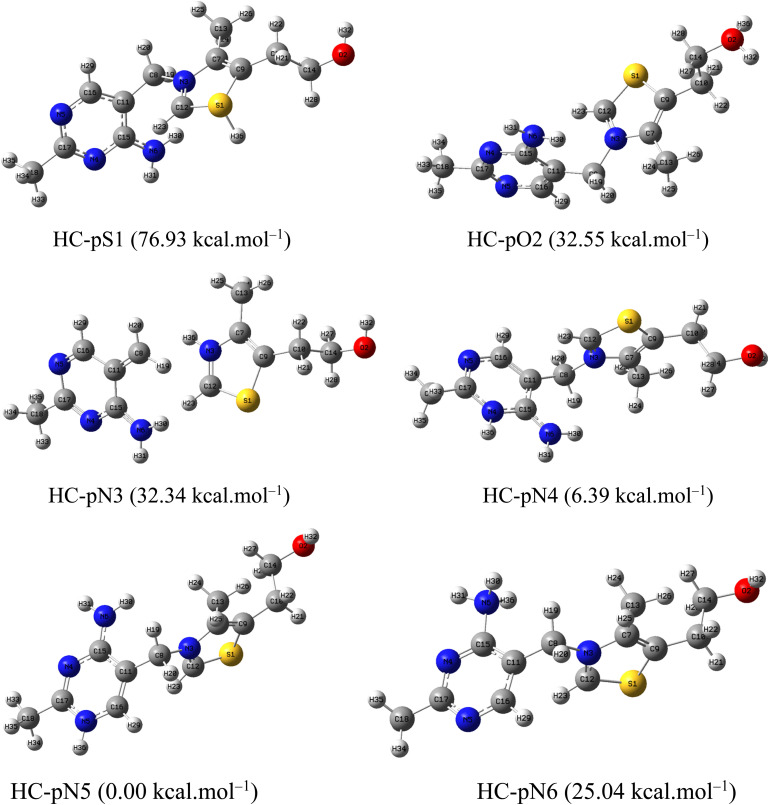
Optimized structures of the protonated forms of HC in aqueous medium.

**Fig. 13:**
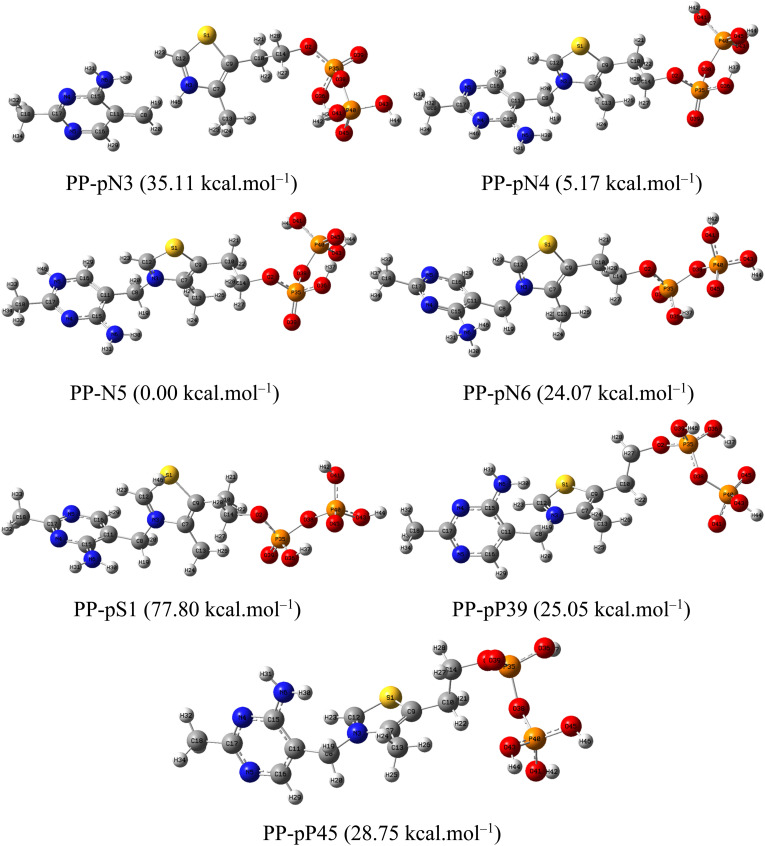
Optimized structures of the protonated forms of PP in aqueous medium.

Since the corrosion experiments were conducted in a strongly acidic environment, the protonated forms of HC and PP were also investigated in order to accurately simulate the molecular states present in solution. For HC, protonation may occur at heteroatom sites possessing lone electron pairs, including S1, O2, N3, N4, N5, and N6 (Fig. 12). In contrast, PP possesses more potential protonation centers, specifically at S1, N3, N4, N5, N6, and the P39 and P45 atoms belonging to the phosphate groups (Fig. 13).

All protonated forms were optimized at the same level of theory, and the relative energy of each structure was determined with respect to the lowest-energy form within each system. The relative energy values (shown in parentheses on the corresponding structures) indicate that HC-pN5 and PP-pN5 possess the lowest energies compared with the other protonated forms. This result demonstrates that protonation at the N5 position represents the most thermodynamically stable state for both molecules under the investigated conditions. Therefore, the HC-pN5 and PP-pN5 structures were selected as representative models for subsequent studies, including the calculation of important electronic parameters such as HOMO and LUMO energies, the energy gap (Δ*E*), global hardness, and molecular softness.

**Fig. 14:**
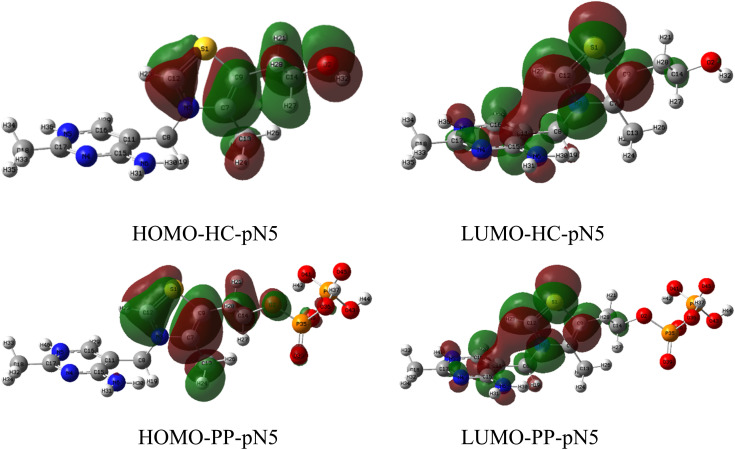
HOMO and LUMO of HC-pN5 and PP-pN5.

The frontier molecular orbital (FMO) distributions of the two molecules HC-pN5 and PP-pN5 were analyzed based on DFT calculations in order to clarify their electron donor–acceptor ability and the mechanism of interaction with the metal surface (Fig. 14).^48^ The results show that the HOMO orbitals of both molecules are mainly localized on the thiazolium ring, with significant contributions from the heteroatoms S1, N3, and O2. The localization of high electron density at these heteroatomic centers indicates that they are strong electron-donating sites, which facilitates electron transfer from the inhibitor molecule to the vacant orbitals of the metal (*e.g.*, the 3d orbitals of Fe), thereby promoting the chemisorption mechanism.

For the LUMO orbitals, both HC-pN5 and PP-pN5 exhibit electron density distributions primarily over the thiazolium ring and partially over the pyrimidine ring. The delocalization of the LUMO over these heterocyclic ring systems indicates the capability for back-donation of electrons from the metal surface to the molecule, particularly through the conjugated π* systems. This suggests that the metal–inhibitor interaction may occur through a bidirectional donor–acceptor electron transfer mechanism.

The important electronic parameters of the two most stable protonated forms, HC-pN5 and PP-pN5, were calculated at the DFT B3LYP/6-311G(d,p) level to evaluate their interaction capability with the metal surface (Table 7). The highest occupied molecular orbital energy (*E*_HOMO_) values of HC-pN5 and PP-pN5 are −7.68 eV and −8.04 eV, respectively. The higher (less negative) *E*_HOMO_ value of HC-pN5 indicates that this molecule tends to donate electrons more readily than PP-pN5, which facilitates the formation of coordination bonds between the heteroatoms (N, S) and the vacant orbitals of Fe on the metal surface.^58^

**Table 7:** Quantum chemical parameters of HC and PP in their protonated forms in an acidic medium

Inhibitors	*E*_HOMO_ (eV)	*E*_LUMO_ (eV)	Δ*E*_L–H_ (eV)	*η* (eV)	*S* (eV^−1^)
HC-pN5	−7.68	−2.55	5.13	2.56	0.39
PP-pN5	−8.04	−2.60	5.44	2.72	0.37

The lowest unoccupied molecular orbital energy (*E*_LUMO_) values of HC-pN5 and PP-pN5 are −2.55 eV and −2.60 eV, respectively. The small difference between these values suggests that the electron back-donation capability from the metal surface to the two molecules is relatively similar. However, considering both *E*_HOMO_ and *E*_LUMO_, the energy gap between the frontier orbitals Δ*E*_L–H_ of HC-pN5 (5.13 eV) is smaller than that of PP-pN5 (5.44 eV). According to frontier molecular orbital theory, a smaller Δ*E*_L–H_ indicates higher chemical reactivity and stronger interaction with the metal surface.^59^ Therefore, the lower Δ*E*_L–H_ value of HC-pN5 suggests that this molecule tends to adsorb more strongly and effectively than PP-pN5.

The global hardness (*η*) values are 2.56 eV for HC-pN5 and 2.72 eV for PP-pN5. The lower *η* value of HC-pN5 indicates that this molecule is less electronically stable and more easily polarized when interacting with the metal surface.^60^

Correspondingly, the molecular softness of HC-pN5 is 0.39 eV^−1^, which is higher than that of PP-pN5 (0.37 eV^−1^). Greater softness reflects a more flexible redistribution of electron density during adsorption, thereby enhancing the interaction between the molecule and the steel surface.^61^

Overall, the electronic parameters indicate that HC-pN5 possesses more favorable electronic characteristics, which is consistent with its stronger adsorption tendency and its more effective corrosion inhibition performance compared with PP-pN5.

### 3.9 Molecular dynamics simulations

Molecular dynamics (MD) simulations were employed to investigate the adsorption behavior of HC-pN5 and PP-pN5 molecules on the Fe (110) surface in a simulated 1.0 M HCl solution environment.^62^ The simulation model consisted of the Fe (110) surface, protonated inhibitor molecules, water molecules, and ions of the acidic solution in order to reproduce the conditions of a corrosive solution. The detailed parameters of the simulation system, including the simulation cell size, the number of molecules, and the computational parameters, are presented in Table S2.

**Fig. 15:**
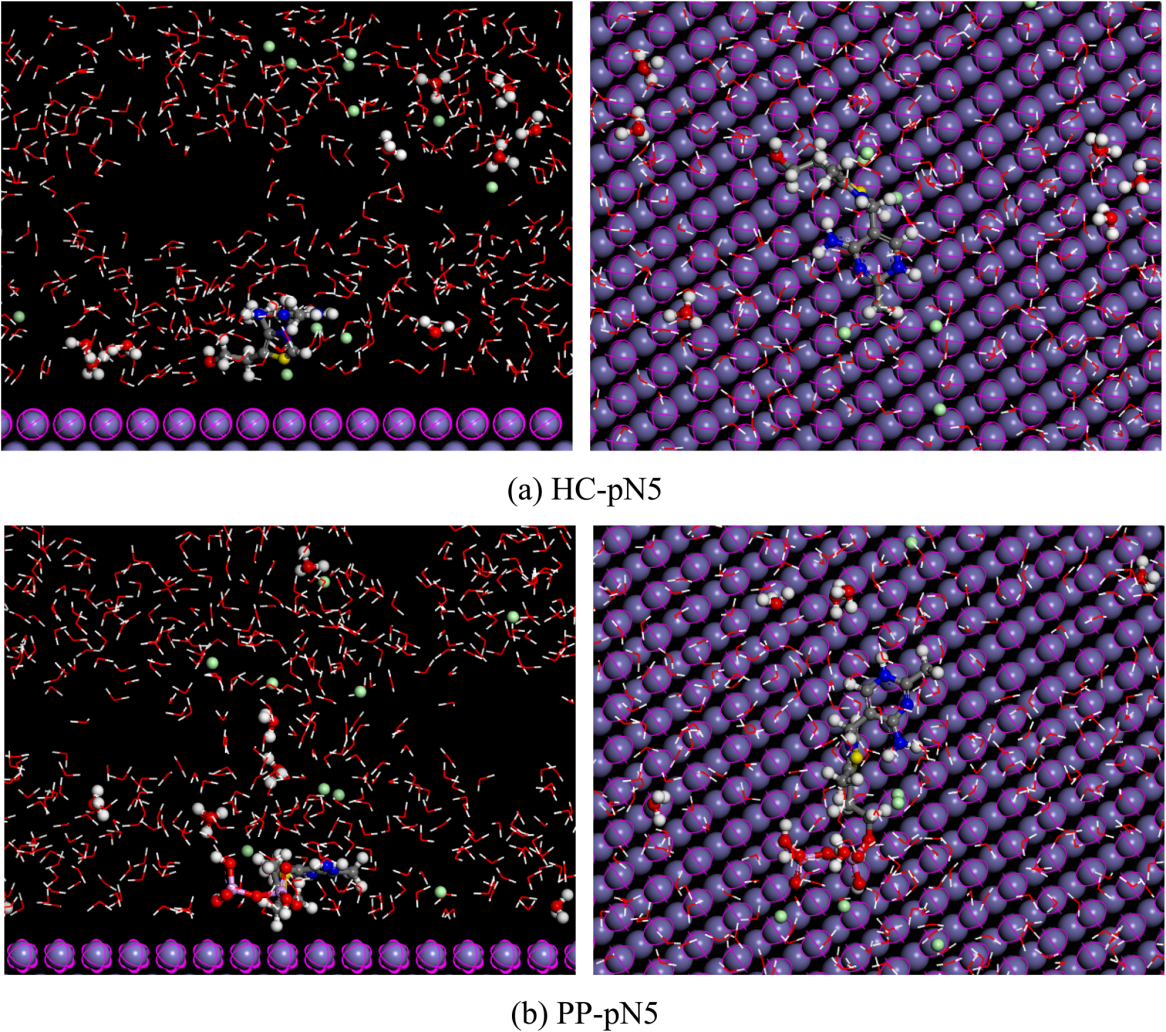
Side and top views of (a) HC-pN5 and (b) PP-pN5 in the simulated environment.

The MD results show that both HC and PP in their protonated forms (HC-pN5 and PP-pN5) tend to adsorb onto the Fe surface with configurations that are nearly parallel to the metal surface. This orientation maximizes the contact area between the inhibitor molecule and the metal surface, facilitating the formation of adsorption interactions between the heteroatoms (such as S, N, and O) as well as the π-electron system of the aromatic rings with the Fe atoms on the metal surface. However, the adsorption configuration of HC-pN5 exhibits a stronger interaction with the surface compared with PP-pN5. Specifically, the HC-pN5 molecule tends to lie closer and spread more extensively across the Fe surface (Fig. 15a), allowing multiple adsorption centers of the molecule to interact simultaneously with the metal surface. As a result, the interactions between the inhibitor molecular orbitals and the Fe d orbitals are intensified through donor–acceptor mechanisms.

In contrast, the PP-pN5 molecule possesses a bulkier structure, leading to the occurrence of steric hindrance, which reduces the likelihood of the molecule coming into close proximity with the metal surface (Fig. 15b). Consequently, the PP-pN5 molecule cannot fully spread over the Fe surface, and fewer adsorption centers participate in interactions with the metal compared with HC. This limitation reduces the effective contact area between PP-pN5 and the Fe surface, resulting in weaker interaction strength. This is clearly reflected in the binding energy values, where PP-pN5 exhibits a value of 272.67 kcal.mol^−1^, which is lower than the 299.32 kcal mol^−1^ obtained for HC-pN5.

These observations imply that HC-pN5 is capable of generating a stable and compact adsorption layer, providing enhanced protection by minimizing metal–environment contact. Therefore, HC-pN5 is predicted to exhibit higher corrosion inhibition efficiency than PP-pN5, which shows good consistency with the experimental results obtained.

### 3.10 Proposed corrosion inhibition mechanism

In a strongly acidic medium (1.0 M HCl), the steel corrosion proceeds through two characteristic electrochemical half-reactions occurring on the metal surface. At the anodic site, iron is oxidized according to the reaction:^63^

12 Fe → Fe^2+^ + 2e

Meanwhile, at the cathodic site, hydrogen ions are reduced to form hydrogen gas:

13 2H^+^ + 2e^−^ → H_2_

The presence of inhibitors in the solution interferes with these processes through a dual adsorption mechanism, including both physical adsorption and chemical adsorption. In the HCl medium, the HC and PP compounds can be readily protonated at the heterocyclic nitrogen atoms, forming the corresponding organic cations (HC-pN5 and PP-pN5). These protonated species carry positive charges while still retaining lone electron pairs on heteroatoms (particularly N and S), allowing them to interact with the metal surface.

The potential of zero charge (PZC, *E*_q=0_) plays a crucial role in determining the nature of the electrostatic interaction between the inhibitor and the metal surface. The surface charge depends on the difference between the corrosion potential and the potential of zero charge, expressed as:

14 *φ* = *E*_corr_ − *E*_q=0_

A negative value *φ* of indicates a negatively charged surface with an affinity for cations, whereas a positive *φ* signifies a positively charged surface that preferentially attracts anions. For iron in HCl solution, *E*_q=0_ is approximately −530 mV *vs.* SCE,^64^ whereas the measured *E*_corr_ is about −325 mV *vs.* SCE. Thus, *φ* = −325 – (−530) = +205 mV > 0, indicating that the steel surface carries a positive charge in 1.0 M HCl. Under these conditions, the adsorption of anions, particularly Cl^−^, is electrostatically favored on the metal surface. The specifically adsorbed Cl^−^ layer forms a locally negatively charged intermediate layer that acts as a charge bridge, thereby facilitating the approach and adsorption of the protonated inhibitor cations (HC-pN5 and PP-pN5) onto the metal surface through electrostatic interactions.

**Fig. 16:**
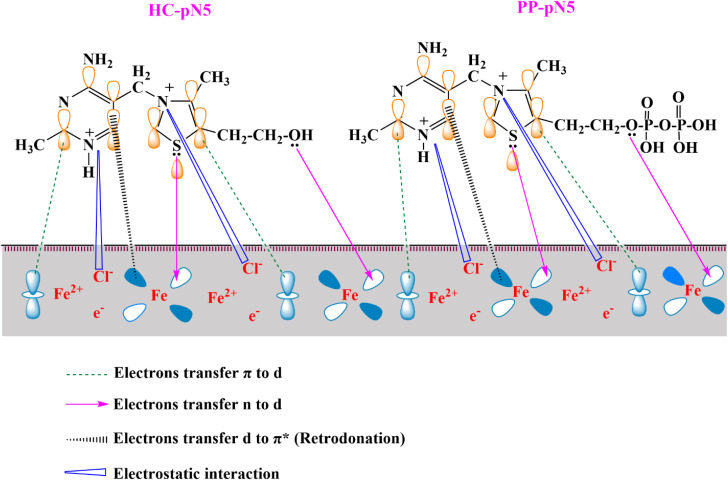
Proposed model for the corrosion inhibition action of HC-pN5 and PP-pN5 on steel in an acidic environment.

After the proton reduction reaction producing H_2_ occurs, a portion of the inhibitor molecules may revert to their neutral state and participate in chemical adsorption. Frontier orbital analysis shows that the HOMO of these compounds is mainly localized on the thiazolium ring and heteroatoms such as S1, O2 and N3, identifying these sites as dominant electron-donating centers. The lone electron pairs on S, O and N can donate electrons to the empty 3d orbitals of iron, leading to the formation of coordinate covalent bonds through donor–acceptor interactions (Fig. 16). This forms the basis of the chemisorption mechanism, enabling the inhibitor molecules to anchor strongly onto the steel surface.

In addition to electron donation from the inhibitor to the metal, a back-donation mechanism may also occur, where electrons from the occupied d orbitals of Fe are transferred back into the π* antibonding orbitals (LUMO) of the inhibitor molecules (Fig. 16).^65^ This bidirectional interaction results in the formation of stable inhibitor–metal complexes, significantly increasing the adsorption energy and strengthening the protective film on the metal surface.

From a structural perspective, the higher inhibition efficiency of HC compared with PP can be explained by differences in steric hindrance. The bulkier structure of PP reduces its ability to approach and orient optimally on the metal surface, thereby limiting surface coverage density and the strength of coordination interactions. In contrast, the more compact structure of HC facilitates a parallel arrangement and the formation of a dense adsorption layer, which enhances the protective efficiency of the steel in acidic environments.

## 4 Conclusion

The corrosion inhibition performance of two thiamine salts, thiamine hydrochloride (HC) and thiamine pyrophosphate (PP), on steel in a hydrochloric acid medium was investigated through a combination of electrochemical techniques and theoretical calculations. The results revealed that both compounds significantly reduced the corrosion rate of steel in the acidic medium; however, HC exhibited higher inhibition efficiency than PP under identical experimental conditions. According to electrochemical impedance spectroscopy measurements, at a concentration of 5 × 10^−3^ M, HC achieved an inhibition efficiency of 87.29%, whereas PP showed an efficiency of 84.10% at room temperature. Potentiodynamic polarization results indicated that both compounds functioned as mixed-type corrosion inhibitors. In addition, the inhibition efficiency of both inhibitors decreased with increasing temperature. At a concentration of 5 × 10^−3^ M, the inhibition efficiency of HC decreased from 91.38% to 80.00% as the temperature increased from 298 K to 323 K, while that of PP decreased from 88.38% to 77.56% over the same temperature range. Analysis of the adsorption behavior of HC and PP molecules on the steel surface indicated that the adsorption process followed the Langmuir isotherm model, whereas the experimental data showed poor agreement with the Temkin model. Surface morphology observations of steel specimens immersed in inhibitor-containing solutions further confirmed that HC provided more effective surface protection than PP in the acidic environment. The formation of the adsorbed inhibitor layer on the steel surface was confirmed by EDX analysis. Moreover, quantum chemical calculations provided additional insight into the corrosion inhibition mechanism at the molecular level. The calculated electronic parameters suggested that the protonated form of HC can establish favorable coordinate interactions with the iron surface, thereby enhancing its adsorption ability on the metal surface. Molecular dynamics simulations further supported this finding, showing that the binding energy of HC-pN5 on the Fe surface was higher than that of PP-pN5 in the simulated environment. Overall, the results obtained from both experimental measurements and theoretical simulations consistently demonstrate that HC acts as a more effective corrosion inhibitor than PP for steel in 1.0 M HCl solution.

## Author contributions

Dinh Quy Huong: funding acquisition, writing – original draft, investigation, data curation, writing – review and editing. Nguyen Minh Tam: data curation. Nguyen Phuc Quynh Ly: visualization, data curation. Le Quoc Thang: supervision, funding acquisition. Pham Cam Nam: methodology, supervision. Dinh Tuan: methodology, investigation. Pham Dinh Tu Tai: conceptualization, funding acquisition.

## Conflicts of interest

There are no conflicts of interest to declare.

## Supplementary Material

RA-016-D6RA02411D-s001

## Data Availability

The data supporting this article have been included as part of the supplementary information (SI). Supplementary information: Fig. S1: showing representative of example simulation of (a) Nyquist and (b) Bode plots recorded for steel in 1.0 M HCl without and with inhibitors; Fig. S2: showing EDX spectra of steel specimens immersed in 1.0 M HCl (a), in the presence of (b) 5 × 10^−3^ M HC and (c) 5 × 10^−3^ M PP; Table S1: presenting details of the EIS parameter calculations; Table S2: presenting details of the molecular dynamics simulations; and Table S3: providing the optimized structures of HC and PP in water at B3LYP/6-311++G(d,p) level. See DOI: https://doi.org/10.1039/d6ra02411d.
