# Supplementary material for: Inhibitory effect of thiamine salts on steel corrosion in an acidic environment: an experimental and theoretical approach
Source: RSC Adv. 2026 May 14;16(28):25365–84. doi: 10.1039/d6ra02411d (PMC13182559; doi:10.1039/d6ra02411d)
Supplement: RA-016-D6RA02411D-s001 [file RA-016-D6RA02411D-s001.pdf]

Supplementary Information (SI) for RSC Advances.
This journal is © The Royal Society of Chemistry 2026

The Supporting Information

Inhibitory Effect of Thiamine Salts on Steel Corrosion in Acidic Environment: An Experimental and Theoretical Approach

Dinh Quy Huong,*[a] Nguyen Phuc Quynh Ly,[a] Pham Dinh Tu Tai,[b] Dinh Tuan,[c] Nguyen Minh Tam,[d] Le Quoc Thang,[a] Pham Cam Nam[e]

[a]Department of Chemistry, University of Education, Hue University, Hue City, Viet Nam

[b]Department of Planning, Finance and Facilities Management, Hue University, Hue City, Viet Nam

[c]Faculty of Pharmacy, Nam Can Tho University, 168 Nguyen Van Cu Ext, Can Tho, Viet Nam

[d]Faculty of Basic Sciences, University of Phan Thiet, 225 Nguyen Thong, Phan Thiet City, Binh Thuan, Viet Nam

[e] Faculty of Chemical Engineering & Strategic Materials & Advanced Research Team – DUT (SMART-DUT), The University of Danang – University of Science and Technology, Danang, 550000, Viet Nam

*: Corresponding author: dqhuong@hueuni.edu.vn

List of supporting information

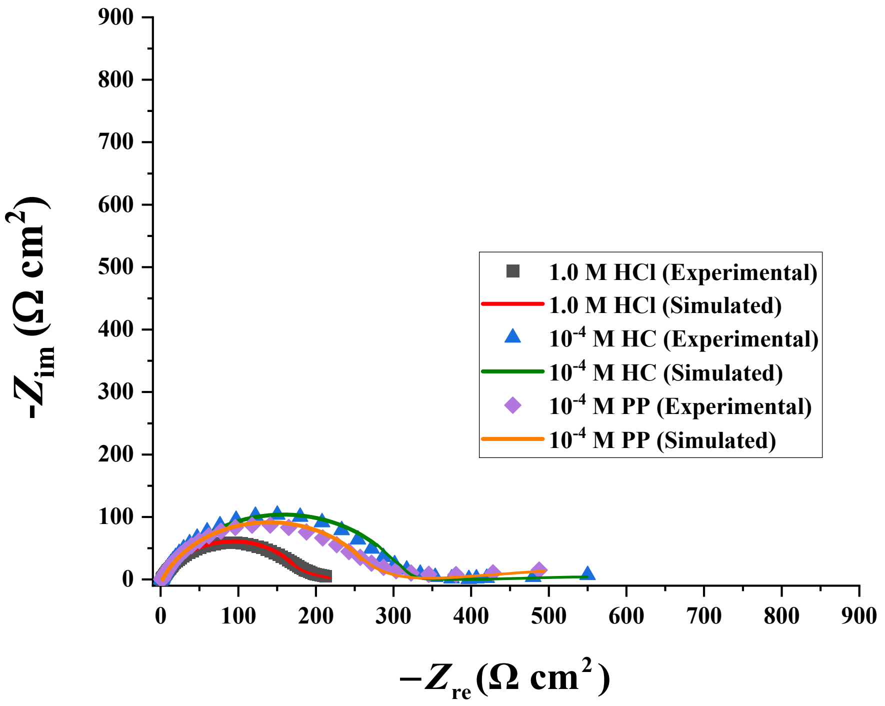

(a)

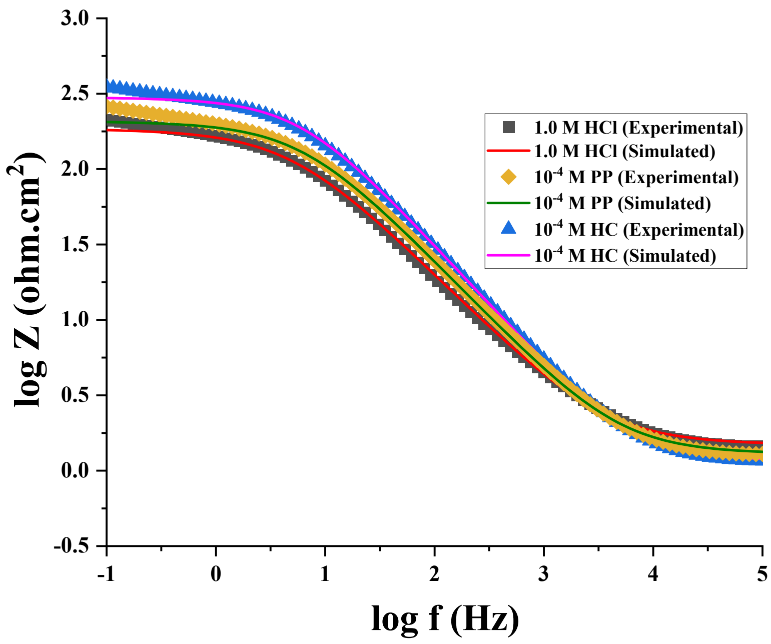

(b)

Fig. S1 A representative of example simulation of (a) Nyquist and (b) Bode plots recorded for steel in 1.0 M HCl without and with inhibitors .

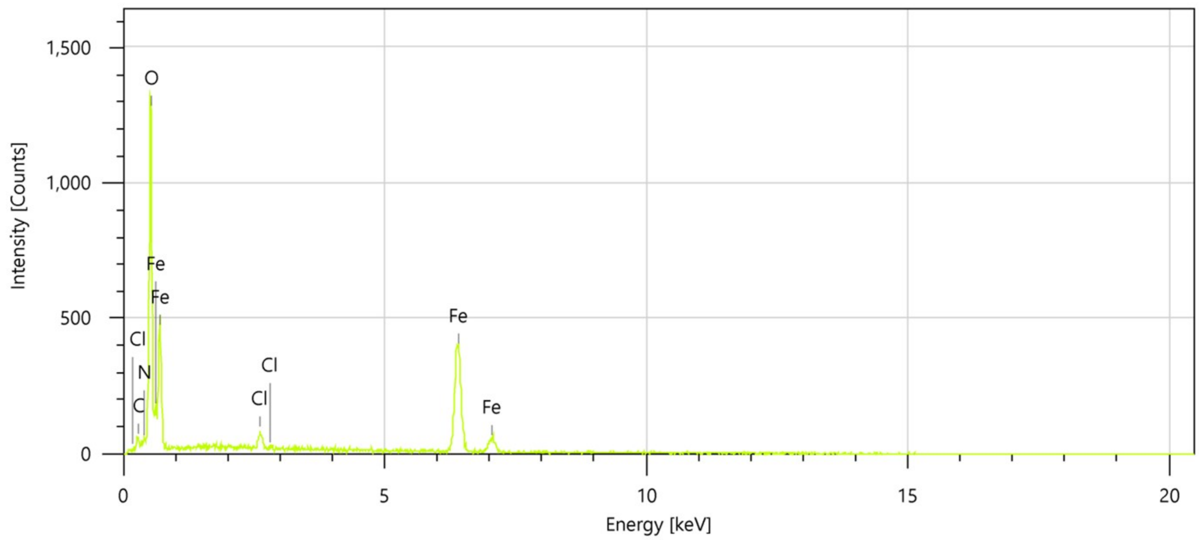

(a) 1.0 M HCl

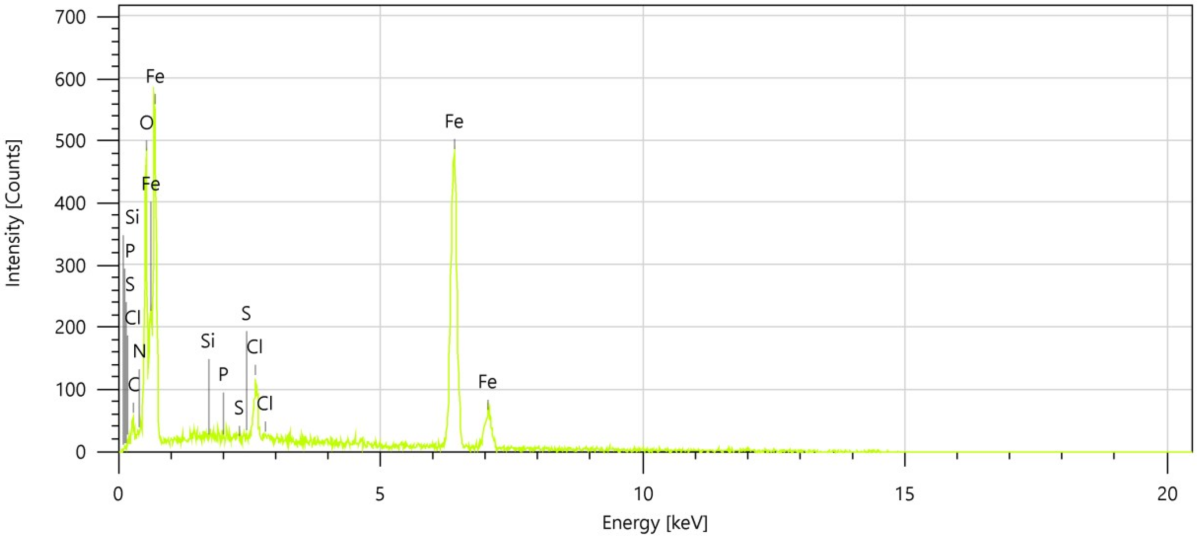

(b) 5×10^{-3} M HC

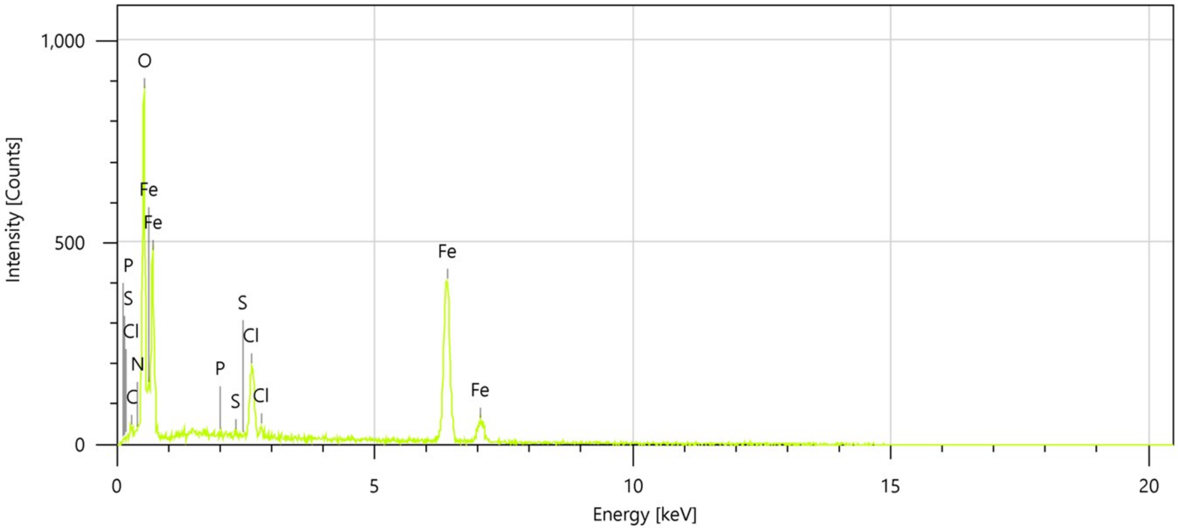

(c) 5×10^{-3} M PP

Fig. S2 EDX spectra of steel specimens (a) in 1.0 M HCl, in the presence of (b) 5×10^{-3} M HC and (c) 5×10^{-3} M PP.

Table S1 Details on the calculation of the EIS parameters

An equivalent circuit model was employed to accurately describe the impedance behavior of the system. In this model, a constant phase element (CPE) is used instead of the ideal double-layer capacitance (C_{dl}) in the classical Randles circuit to account for surface heterogeneity and non-ideal capacitive behavior at the electrode/electrolyte interface. The impedance of the CPE is defined as follows: $$Z_{CPE} = Y_o^{-1}.(j\omega)^{-n} \quad (1')$$ Where ω is the angular frequency ($\omega = 2\pi f$, f is the frequency), j is the virtual unit, Y_o is the value of CPE and n is the compression ratio. n equals 1 when the electrode surface is homogeneous and flat. The value of C_{dl} can be estimated according to the equation: $$C_{dl} = Y_o.(\omega_{max})^{n-1} \quad (2')$$ where ω_{max} is the angular frequency at which the imaginary component of the impedance reaches its maximum value, defined as: $$\omega_{max} = 2\pi f_{max} \quad (3')$$ This approach provides a more realistic estimation of the interfacial capacitance and enables a more accurate interpretation of the electrochemical processes occurring at the electrode surface.

Table S2 Details on molecular dynamics simulations

Molecular dynamics (MD) simulations were carried out using Materials Studio 8.0 developed by BIOVIA Inc.. The interaction between the inhibitor molecule and the steel surface was investigated within a simulation box of dimensions 30.40 × 38.20 × 60.25 Å. Under ambient conditions, iron crystallizes in a body-centered cubic (bcc) structure, exposing several low-index surfaces such as (110), (100), (111), (211), (311), (321), and (210). Among these, the Fe(110) facet is the most thermodynamically stable and exhibits the highest surface atomic density, thereby contributing dominantly to the overall crystal morphology. Consequently, the Fe(110)

surface was selected as the representative adsorption substrate for modeling the inhibitor–metal interaction in an aggressive medium.

The simulation cell was constructed with periodic boundary conditions, comprising a lower Fe(110) slab and an upper aqueous phase. In the simulated corrosive environment, the composition of the aqueous phase was established based on a molar ratio of H_2O to HCl of 500:9, in order to realistically reproduce acidic conditions at the molecular level. Accordingly, the baseline simulation system was constructed to include one inhibitor molecule, 491 water molecules, 9 hydronium ions (H_3O^+), and 9 chloride ions (Cl^-), ensuring both chemical representativeness and overall charge neutrality.

For systems involving protonated inhibitor species, slight adjustments in the composition were required to maintain electrostatic balance. In the case of protonated HC, the system consisted of one protonated HC cation, 491 water molecules, 9 hydronium ions, and 11 chloride ions. The increased number of chloride ions compensates for the additional positive charge introduced by protonation of the inhibitor molecule. Similarly, for the protonated PP system, the composition was defined as one protonated PP cation, 492 water molecules, 8 hydronium ions, and 10 chloride ions. This modification reflects a redistribution of ionic species to preserve electroneutrality while maintaining a composition close to the target H_2O/HCl molar ratio.

The system was equilibrated at 298 K under the canonical NVT ensemble for a total simulation time of 1000 ps, with an integration time step of 1.0 fs, employing the COMPASS II force field to accurately describe interatomic interactions. Long-range electrostatic interactions as well as van der Waals forces were treated using the Ewald summation method to ensure computational accuracy.

Prior to MD simulations, the geometrical optimization of the protonated inhibitor molecules was performed using the DMol3 package at the density functional theory (DFT) level, with the B3LYP exchange–correlation functional and a DNP basis set, along with a global orbital cutoff of 3.5 Å.<br><br>The interaction between the inhibitor molecule and the Fe(110) surface was quantified in terms of the interaction energy ($E_{interaction}$), calculated using the following expression:<br><br>$$E_{interaction} = E_{total} - (E_{surface+solution} + E_{inhibitor}) \quad (4')$$<br><br>where E_{total} represents the total energy of the entire simulation system, $E_{surface+solution}$ corresponds to the energy of the Fe(110) slab together with water molecules, hydronium ions, and chloride ions, and $E_{inhibitor}$ is the energy of the isolated inhibitor molecule. Furthermore, the binding energy ($E_{binding}$) is directly related to the interaction energy and can be expressed as follows:<br><br>$$E_{binding} = -E_{interaction} \quad (5')$$<br><br>A higher (more positive) binding energy indicates stronger adsorption of the inhibitor on the metal surface, reflecting enhanced corrosion inhibition performance.

Table S3 Optimized structure of HC and PP in water using B3LYP/6-311++G(d,p)

**HC**	$[C_{12}H_{17}N_4OS]^+$
1 1<br>S -1.78517100 -1.58969500 -0.91898200<br>O -6.10046700 -0.52860300 0.93053200<br>N -0.43797400 0.47728700 -0.45392000<br>N 3.70154000 -0.43945400 1.14013500<br>N 4.19247500 0.19313500 -1.10437400	

N	1.68997500	-0.00140100	2.14587100
C	-1.76554800	0.90253800	-0.32823600
C	0.72714600	1.41674100	-0.30430600
C	-2.64722000	-0.11595100	-0.55456000
C	-4.14368100	-0.10494900	-0.47736700
C	2.04891400	0.73312300	-0.15086400
C	-0.30206200	-0.80114300	-0.76368500
C	-2.08172400	2.32319600	0.00926100
C	-4.68070500	-0.49363300	0.91636400
C	2.47454800	0.10921100	1.05179800
C	2.97579000	0.73725300	-1.18325400
C	4.49715900	-0.37819600	0.07334300
C	5.86566600	-0.98711700	0.19494800
H	0.50113000	2.05087300	0.55331100
H	0.73055800	2.05109300	-1.19112800
H	-4.56377500	-0.79427800	-1.21326300
H	-4.50648600	0.89298300	-0.73637300
H	0.65442300	-1.27618500	-0.90881900
H	-1.73745400	2.57941400	1.01470800
H	-1.61700100	3.01326800	-0.69854100
H	-3.15731300	2.48293100	-0.02329100
H	-4.28763700	0.19396700	1.67403600
H	-4.34798400	-1.50136500	1.17317800

H	2.71797600	1.21338800	-2.12663700	
H	0.81711600	0.48942600	2.24210800	
H	2.10917200	-0.37160500	2.98485000	
H	-6.42331200	0.37769100	0.87754700	
H	5.96529400	-1.53132200	1.13235800	
H	6.05425400	-1.65870200	-0.64552000	
H	6.62569000	-0.20103700	0.15631300	
**PP**				$[C_{12}H_{19}N_4O_7P_2S]^+$
1 1				
S	-1.22640100	0.40569900	2.03728200	
O	3.15714600	-1.36629100	0.97180100	
N	-2.35595000	0.39545800	-0.20613100	
N	-6.68461700	-0.95409400	0.02591600	
N	-6.91372600	1.40100900	-0.25533100	
N	-4.73116400	-2.07533200	-0.39444100	
C	-0.99510200	0.29238900	-0.51248900	
C	-3.41573700	0.46636600	-1.27179000	
C	-0.22431500	0.28572800	0.61447700	
C	1.26692000	0.16645100	0.72858800	
C	-4.81354700	0.33485100	-0.75502600	
C	-2.61930600	0.46604800	1.08787900	
C	-0.53401200	0.21638300	-1.93134600	
C	1.72053000	-1.29862100	0.77130200	

C	-5.40391300	-0.90352300	-0.38802600
C	-5.64459000	1.44285200	-0.66684000
C	-7.37557600	0.18354500	0.07806500
C	-8.80569600	0.10333100	0.53199400
H	-3.17356800	-0.31426900	-1.99354100
H	-3.28771000	1.42733400	-1.77074200
H	1.61423600	0.66890200	1.63348700
H	1.73432200	0.67697400	-0.11541200
H	-3.61741400	0.56311500	1.48293900
H	-0.87924600	-0.70176700	-2.41361800
H	-0.89745400	1.06682900	-2.51270300
H	0.55327500	0.22314700	-1.97113600
H	1.45404600	-1.83163700	-0.14203700
H	1.28842700	-1.81816100	1.62462700
H	-5.25903500	2.41983900	-0.94967800
H	-3.83325200	-2.18060100	-0.83562300
H	-5.26162800	-2.91554400	-0.22224800
H	-9.02731700	-0.88159700	0.93919300
H	-9.00633900	0.87086500	1.28225600
H	-9.47318400	0.29553300	-0.31364900
P	4.19597200	-1.47848600	-0.23357800
O	5.53858700	-1.90348800	0.48054600
H	6.04627100	-1.13851000	0.84892900

O	4.44359300	0.08768200	-0.68655600
O	3.77147100	-2.29301800	-1.38141900
P	5.44768400	1.20533400	-0.09921800
O	4.54001500	2.40293600	0.41635800
H	4.31057400	2.35981900	1.35557500
O	6.03572900	1.75621100	-1.46142600
H	6.64448100	2.50355000	-1.37586200
O	6.37625500	0.61699200	0.90242500

Table S4 Optimized structures of protonated HC and PP in water using B3LYP/6-311G(d,p)

HC-pS1			$[C_{12}H_{18}N_4OS]^{2+}$
2 1			
S	-1.77294700	-1.78391300	-0.57028200
O	-6.26389900	-0.18743800	0.73763100
N	-0.42649600	0.34408500	-0.35787500
N	3.87854300	-0.23440500	1.09805600
N	4.12736700	0.10498100	-1.24857900
N	1.97389700	0.30198500	2.24592300
C	-1.79802800	0.78650300	-0.41354600
C	0.72558900	1.34379500	-0.29038400
C	-2.68512400	-0.22921700	-0.45599400
C	-4.17568500	-0.22130300	-0.51226300
C	2.06938100	0.71617500	-0.16090500

C	-0.23616400	-0.92588000	-0.36751700
C	-2.10602400	2.24218200	-0.41643400
C	-4.85832100	-0.14214600	0.88139900
C	2.63172600	0.27707800	1.06888100
C	2.89665400	0.60722700	-1.27615500
C	4.56217300	-0.30475400	-0.04048300
C	5.94864300	-0.87666400	0.01547200
H	0.46968800	2.00655900	0.53672200
H	0.64795100	1.91372200	-1.21626700
H	-4.54324200	-1.11110300	-1.02519200
H	-4.48384400	0.64006600	-1.11052300
H	0.72098800	-1.42717400	-0.32821500
H	-1.79234400	2.71046300	0.51938100
H	-1.60870400	2.74760200	-1.24655900
H	-3.17825300	2.38981900	-0.52228200
H	-4.52973900	0.76004600	1.40680300
H	-4.58130200	-1.00726500	1.48418700
H	2.53306800	0.95280400	-2.24134200
H	1.11502900	0.80747200	2.38513500
H	2.49664600	0.05001400	3.07116600
H	-6.56646700	0.66572400	0.40685000
H	6.17147200	-1.25668100	1.01057300
H	6.05126500	-1.67850300	-0.71950500

H	6.67721000	-0.10447800	-0.24773400	
H	-1.84317500	-2.27149000	0.70516800	
HC-pO2				$[C_{12}H_{18}N_4OS]^{2+}$
2 1				
S	-1.73570800	-1.64917300	-0.77531500	
O	-6.09978300	-0.42613000	0.98380600	
N	-0.38557600	0.45387400	-0.50798700	
N	3.75067100	-0.29791600	1.19084500	
N	4.22391800	0.02196300	-1.12262200	
N	1.76327200	0.31249900	2.15424400	
C	-1.71039200	0.89206400	-0.40831700	
C	0.78759100	1.39847100	-0.45626900	
C	-2.58904700	-0.14613000	-0.53182500	
C	-4.09200000	-0.11267700	-0.48344100	
C	2.10202100	0.72265600	-0.22621300	
C	-0.25576900	-0.84770500	-0.70146900	
C	-2.01753000	2.33924500	-0.20595300	
C	-4.58867700	-0.43615900	0.92167900	
C	2.53438600	0.25810900	1.04544300	
C	3.01535700	0.57485800	-1.25954500	
C	4.53169600	-0.39916400	0.11514000	
C	5.86880800	-1.05857300	0.30104100	
H	0.56078500	2.12329900	0.32526400	

H	0.79679300	1.93103500	-1.40765100	
H	-4.50016300	-0.83225900	-1.19613300	
H	-4.43897200	0.87601300	-0.78760500	
H	0.69901500	-1.33697300	-0.80747900	
H	-1.64043000	2.69170900	0.75753400	
H	-1.57181400	2.94960500	-0.99441700	
H	-3.09246100	2.50476800	-0.22056000	
H	-4.30684200	0.29731600	1.67053300	
H	-4.33896300	-1.43757100	1.25994800	
H	2.75532600	0.92971900	-2.25422500	
H	0.92264400	0.86358200	2.20094600	
H	2.19735300	0.06362900	3.02999200	
H	-6.51491200	0.44550200	0.84174300	
H	6.21946900	-0.93778200	1.32556600	
H	5.77861900	-2.13148000	0.09973100	
H	6.59641900	-0.64919700	-0.39957000	
H	-6.53694100	-1.09803100	0.42799400	
HC-pN3				$[C_{12}H_{18}N_4OS]^{2+}$
2 1				
S	-5.23761300	-1.05780200	-1.55639700	
O	-8.20486700	-1.15860900	2.20614000	
N	-3.93014000	1.03600100	-1.22094600	
N	7.37798000	-1.08787400	-0.11317600	

N	7.78022900	1.29116300	0.02416700
N	5.37122900	-2.00367500	0.40224900
C	-5.03649800	1.22686700	-0.40644600
C	4.42081300	0.69378800	1.24224100
C	-5.88134200	0.15477200	-0.46909000
C	-7.15989800	-0.08082900	0.27350500
C	5.62756900	0.41257000	0.70834800
C	-3.88648400	-0.10290300	-1.88875300
C	-5.16271600	2.49051400	0.37967900
C	-6.96274200	-0.88749900	1.57374200
C	6.10925700	-0.92244300	0.33410900
C	6.58706300	1.48015300	0.47660200
C	8.14114500	-0.02852500	-0.24404300
C	9.53322900	-0.20466800	-0.73005000
H	3.67310900	-0.05705500	1.46641400
H	4.15933300	1.71856700	1.48106400
H	-7.87314100	-0.61392600	-0.35890500
H	-7.60868900	0.88614400	0.51440800
H	-3.08589700	-0.36911100	-2.56051000
H	-4.37734800	2.55362800	1.13698600
H	-5.07563200	3.36048900	-0.27560800
H	-6.12654100	2.53443300	0.88259200
H	-6.27701700	-0.35528900	2.24286600

H	-6.51852400	-1.85793400	1.34316400
H	6.27864500	2.49750500	0.70617300
H	4.40651700	-2.00559200	0.69859200
H	5.78635500	-2.88643900	0.13008600
H	-8.54410400	-0.33222900	2.56758400
H	9.76152500	-1.25184600	-0.90992800
H	9.66601300	0.37694500	-1.64804500
H	10.22595700	0.21821500	0.00406000
H	-3.20128100	1.73713500	-1.31288100

HC-pN4	$[C_{12}H_{18}N_4OS]^{2+}$

2 1			
S	-1.85092300	-1.86129300	-0.13960800
O	-6.14302600	-0.02984900	1.07945400
N	-0.48390600	0.19274600	-0.60105900
N	3.73842100	0.18370500	1.10422100
N	4.13094500	-0.40335600	-1.11780900
N	1.78480900	1.12954300	1.90375400
C	-1.80787400	0.64456800	-0.67067500
C	0.67123500	1.10171500	-0.88062300
C	-2.69759200	-0.36535800	-0.43839100
C	-4.19335400	-0.30606100	-0.37224400
C	2.00761300	0.56335500	-0.45900600
C	-0.36080500	-1.10028700	-0.33853900

C	-2.11334700	2.07350300	-0.98177600
C	-4.72446200	0.01188300	1.04186500
C	2.47541500	0.64545500	0.88345600
C	2.88687900	0.05063100	-1.38772500
C	4.53817100	-0.33425900	0.12337800
C	5.88938000	-0.81726300	0.52753200
H	0.45684600	2.04976200	-0.38828100
H	0.67710600	1.28814500	-1.95428500
H	-4.62168300	-1.25771900	-0.69399200
H	-4.55099300	0.45416500	-1.07149100
H	0.58625700	-1.60930500	-0.26775400
H	-1.78293300	2.73823600	-0.17921700
H	-1.63445200	2.39064800	-1.91095800
H	-3.18704600	2.20410700	-1.09838100
H	-4.34079700	0.98217500	1.37782800
H	-4.37787600	-0.74726500	1.74556500
H	2.58548800	-0.00399400	-2.42865000
H	0.84869100	1.48404600	1.78826300
H	2.16833100	1.17948300	2.83722600
H	-6.47968000	0.73181500	0.59476100
H	5.80101000	-1.64378400	1.23843400
H	6.42171800	-1.15716900	-0.35667300
H	6.45615800	-0.01760300	1.01121300

H	4.11337200	0.22797400	2.04722700	
HC-pN5				$[C_{12}H_{18}N_4OS]^{2+}$
2 1				
S	-1.86922100	-1.85138000	-0.27392200	
O	-6.09243100	-0.06144300	1.17507600	
N	-0.47420300	0.19827300	-0.66957200	
N	3.65829400	0.16121500	1.26892600	
N	4.15326300	-0.32931800	-0.94798800	
N	1.64278000	1.02391100	1.87128000	
C	-1.79056500	0.67979600	-0.66497800	
C	0.69488900	1.08320700	-0.96851000	
C	-2.69332600	-0.32452600	-0.45980700	
C	-4.18526400	-0.24383200	-0.34576300	
C	2.01263700	0.55140700	-0.48224400	
C	-0.37142700	-1.11035500	-0.48350700	
C	-2.07680800	2.13105900	-0.87646200	
C	-4.67453900	-0.04470200	1.10449800	
C	2.42391000	0.58576400	0.89426800	
C	2.93628200	0.08411900	-1.37227600	
C	4.49571800	-0.28235700	0.37044500	
C	5.86055500	-0.74306700	0.75596400	
H	0.48415400	2.05265900	-0.51926200	
H	0.72329500	1.22425000	-2.04894100	

H	-4.64108800	-1.15485100	-0.73965000
H	-4.54457500	0.58189900	-0.96497700
H	0.56647400	-1.64134900	-0.47860100
H	-1.68420000	2.73934900	-0.05728500
H	-1.64522300	2.49245000	-1.81265200
H	-3.15178600	2.29196000	-0.92021000
H	-4.26157100	0.88336300	1.51597600
H	-4.32788700	-0.87124500	1.72781400
H	2.75122500	0.02447400	-2.43588900
H	0.70843500	1.37003700	1.72574000
H	2.00661600	1.03210400	2.81392700
H	-6.42364900	0.74930500	0.77327200
H	5.95817700	-0.71179800	1.83781300
H	6.03626900	-1.76090900	0.40008100
H	6.61624000	-0.09430100	0.30477800
H	4.82275600	-0.67140600	-1.62808400
HC-pN6		$[C_{12}H_{18}N_4OS]^{2+}$	
2 1			
S	-1.84830100	-1.80545000	0.41772400
O	-6.23780500	0.16190700	0.87012500
N	-0.48995300	0.09554100	-0.50090900
N	3.92282300	0.46102500	0.93682900
N	3.95024200	-0.86300100	-1.02237700

N	2.18826100	1.85052900	1.60680300
C	-1.81551300	0.47375400	-0.75062000
C	0.65146500	0.95777200	-0.93375600
C	-2.70044300	-0.46729800	-0.30756800
C	-4.19820600	-0.43854000	-0.34083000
C	2.00752400	0.46731700	-0.50363600
C	-0.36152500	-1.07577800	0.10464800
C	-2.12458300	1.76579300	-1.43318600
C	-4.81974300	0.21688000	0.91125900
C	2.71853200	0.87019200	0.62435900
C	2.73073600	-0.42046300	-1.31278500
C	4.51512700	-0.41579800	0.10493000
C	5.88059400	-0.89731400	0.47297700
H	0.44334700	1.96014100	-0.56467600
H	0.61793400	1.00193400	-2.02236200
H	-4.59203000	-1.45312200	-0.42983500
H	-4.52273300	0.10474900	-1.23186600
H	0.58585100	-1.51731600	0.36523200
H	-1.80023500	2.62072200	-0.83414900
H	-1.64109800	1.82697800	-2.41121600
H	-3.19787500	1.85660400	-1.58523400
H	-4.45953600	1.24685300	1.01392400
H	-4.51812300	-0.33382800	1.80434100

H	2.29056400	-0.77743600	-2.23936000
H	1.88725700	2.73213300	1.17825400
H	2.95243400	2.06577500	2.25826500
H	-6.54365000	0.78080100	0.19791400
H	5.83909600	-1.43625200	1.42341400
H	6.27206200	-1.55398700	-0.30050200
H	6.55174500	-0.04656700	0.61265500
H	1.40685000	1.48600000	2.16343500

PP-pS1	$[C_{12}H_{20}N_4O_7P_2S]^{2+}$

2 1

S	-1.47122600	2.16611400	0.61316900
O	3.14118500	0.19629100	1.07227100
N	-2.59793500	0.14002900	-0.39741800
N	-6.95838600	-0.62983500	0.84190100
N	-7.13467300	0.53768400	-1.22942800
N	-5.03412100	-1.64625600	1.54436400
C	-1.18562300	-0.11486800	-0.54974700
C	-3.64460700	-0.81017300	-0.97986800
C	-0.41304200	0.84146500	0.00292800
C	1.07507100	0.96351500	0.03923400
C	-5.04481100	-0.47664900	-0.60005400
C	-2.91657900	1.21566100	0.22575600
C	-0.72421400	-1.33858500	-1.25841000

C	1.70738400	0.24645700	1.24912900
C	-5.66785800	-0.93042700	0.59548300
C	-5.86101700	0.24009200	-1.47166600
C	-7.62539500	0.08706300	-0.05778400
C	-9.05244000	0.43300400	0.25291300
H	-3.33269300	-1.80350900	-0.66005500
H	-3.49576600	-0.74085000	-2.05786300
H	1.37698000	2.01121300	0.03362600
H	1.45770800	0.51086300	-0.87716200
H	-3.92189000	1.55618800	0.43361800
H	-1.08765500	-2.23747500	-0.75455100
H	-1.08170700	-1.34690600	-2.29099700
H	0.36413900	-1.37826300	-1.26501800
H	1.30644000	-0.76039100	1.36228800
H	1.54888300	0.80110200	2.17182800
H	-5.45276600	0.58467100	-2.41920300
H	-4.12324900	-2.05148000	1.40992300
H	-5.58811200	-1.99579200	2.31129300
H	-9.40322500	-0.11811100	1.12348600
H	-9.13395700	1.50554100	0.45488300
H	-9.68813100	0.21786300	-0.60820000
P	3.82378900	-0.94522200	0.17486000
O	4.66745700	-1.86604100	1.14618800

H	5.54454600	-1.48099100	1.39007400
O	4.93729600	-0.06479100	-0.63068000
O	2.89287400	-1.65786500	-0.71569400
P	6.45219100	0.34981500	-0.24016300
O	6.48391400	1.93631200	-0.22599400
H	6.30094300	2.33715300	0.63569900
O	7.16821100	-0.01715900	-1.60146300
H	8.11598100	0.17527700	-1.63562700
O	6.88110100	-0.32627500	1.01129400
H	-1.48828000	1.93271300	1.96001500

PP-pN3	$[C_{12}H_{20}N_4O_7P_2S]^{2+}$

2 1			
S	-0.55924300	2.79247400	1.04765100
O	4.07068900	1.57813600	0.91288800
N	-1.59714800	1.33501100	-0.68526700
N	-8.19113200	-0.69301400	0.80616600
N	-7.58527600	-0.42840400	-1.51824500
N	-6.75146600	-1.52189900	2.34624100
C	-0.24852900	1.27854500	-0.99017700
C	-4.66914300	-1.70656000	0.20538700
C	0.48041900	2.02872400	-0.13327400
C	1.96666200	2.23382100	-0.10080800
C	-5.94124200	-1.27773400	0.03986100

C	-1.92405500	2.08479400	0.35243900
C	0.20429800	0.46716200	-2.15084300
C	2.66044100	1.26158200	0.85625300
C	-6.95769700	-1.17158000	1.09764700
C	-6.40259200	-0.86982700	-1.27703700
C	-8.45635300	-0.34965800	-0.43235900
C	-9.80737600	0.17378100	-0.75787400
H	-4.25906200	-2.01413100	1.15900800
H	-3.99893200	-1.76177100	-0.64075600
H	2.20000200	3.25566500	0.20599700
H	2.36199300	2.10136900	-1.10942400
H	-2.93911500	2.21711000	0.69728200
H	-0.11479200	-0.57197000	-2.03962000
H	-0.22391400	0.86130600	-3.07529600
H	1.28986100	0.48325500	-2.23788200
H	2.51587900	0.22688200	0.55486300
H	2.30199600	1.38096300	1.87777100
H	-5.69940700	-0.93888100	-2.10514700
H	-5.87972700	-1.89898700	2.68663900
H	-7.50905700	-1.40743500	3.00945500
H	-10.44766500	0.19351200	0.11978400
H	-9.70910300	1.17937800	-1.18061500
H	-10.25201100	-0.44679800	-1.54276500

P	5.20259400	0.81215600	0.11971800
O	4.67849300	0.68901500	-1.38460100
H	4.84286800	-0.19788400	-1.78543700
O	5.07414600	-0.72714900	0.70107900
O	6.52910700	1.40634300	0.33815800
P	5.30073700	-2.13077900	-0.05865500
O	4.29302600	-3.13243000	0.64104500
H	3.40466200	-3.15503200	0.25101300
O	6.71230400	-2.55425900	0.52600400
H	7.09521600	-3.35522500	0.14436900
O	5.16311600	-1.96606800	-1.52698700
H	-2.30266900	0.83956100	-1.22584300

PP-pN4	$[C_{12}H_{20}N_4O_7P_2S]^{2+}$

2 1

S	-1.15183500	1.08722700	1.81411300
O	3.11137800	-1.12434900	1.14120500
N	-2.35155800	0.54574400	-0.32424500
N	-6.58293800	-1.00130700	0.17685600
N	-6.98992100	1.22912900	-0.36660900
N	-4.57556300	-2.10392400	-0.14093000
C	-1.00307500	0.31852200	-0.62682000
C	-3.43492800	0.39741200	-1.34686600
C	-0.20035000	0.56477300	0.45016300

C	1.28744400	0.40397700	0.56395100
C	-4.81165100	0.24374600	-0.76799100
C	-2.57012300	0.96081700	0.91560000
C	-0.58412400	-0.12556400	-1.99025900
C	1.67657800	-1.03177700	0.94412000
C	-5.28691600	-0.99206000	-0.24365200
C	-5.71287000	1.28528300	-0.80587100
C	-7.40703200	0.08848100	0.11925000
C	-8.79679400	-0.07900400	0.63150500
H	-3.17192900	-0.45857600	-1.96757600
H	-3.40313400	1.28256200	-1.98149400
H	1.68196200	1.08648700	1.31914900
H	1.75074700	0.68037800	-0.38512300
H	-3.54818100	1.19488700	1.30302700
H	-0.99733500	-1.10691600	-2.23618700
H	-0.90294900	0.58503400	-2.75638600
H	0.49994800	-0.20322900	-2.03773500
H	1.36845300	-1.74935900	0.18315400
H	1.24010100	-1.31688900	1.89973300
H	-5.40257100	2.23970000	-1.21843600
H	-3.61135400	-2.14736600	-0.43055400
H	-4.96757400	-2.95684400	0.23369600
H	-8.78309700	-0.36924800	1.68548200

H	-9.32996700	0.86129900	0.52091500
H	-9.31839600	-0.86188100	0.07444400
P	4.12422000	-1.52116400	-0.02695700
O	5.42152400	-1.98236300	0.74719800
H	5.98913000	-1.22854000	1.04150400
O	4.53630700	-0.07132600	-0.69354000
O	3.60599000	-2.44083800	-1.04978500
P	5.60529800	1.03361700	-0.20138600
O	4.77704700	2.34943100	0.12655800
H	4.52083900	2.44427400	1.05484400
O	6.27933200	1.36708000	-1.59409100
H	6.94190600	2.07207400	-1.57165600
O	6.45056200	0.50733900	0.90287000
H	-6.96421400	-1.86520000	0.55094200

PP-pN5	$[C_{12}H_{20}N_4O_7P_2S]^{2+}$

2 1

S	1.17052600	-0.47107700	2.04964400
O	-3.14484100	1.27799000	0.98761200
N	2.36461200	-0.56709300	-0.15749100
N	6.61976100	1.11754800	-0.12073800
N	6.97594900	-1.17876200	-0.07210200
N	4.60307400	1.99376600	-0.70002300
C	1.01232500	-0.46836100	-0.50963300

C	3.44003600	-0.72237800	-1.18633500
C	0.21128600	-0.40432300	0.59406500
C	-1.28058200	-0.26478600	0.66540500
C	4.82337700	-0.42592400	-0.68368300
C	2.58847700	-0.58576300	1.14850500
C	0.59237700	-0.45717400	-1.94313400
C	-1.71420900	1.20422700	0.75565400
C	5.33453500	0.90531000	-0.50589500
C	5.70576000	-1.44306900	-0.45711300
C	7.41368900	0.10212000	0.08853400
C	8.83213600	0.30805500	0.49993700
H	3.18381700	-0.06392000	-2.01565100
H	3.38664500	-1.74669300	-1.55367500
H	-1.66342600	-0.79599800	1.53926700
H	-1.72738300	-0.73363600	-0.21269900
H	3.56937400	-0.67572900	1.58561000
H	0.95810200	0.43393300	-2.45972800
H	0.95953300	-1.33917200	-2.47272700
H	-0.49328000	-0.45443100	-2.01157100
H	-1.45879200	1.75969500	-0.14724100
H	-1.25920000	1.69435300	1.61467600
H	5.44560700	-2.48614900	-0.57494000
H	3.63944600	1.97568400	-0.99313700

H	5.03872400	2.89638700	-0.57082600
H	9.00833800	1.36695900	0.66799800
H	9.05173900	-0.25218500	1.41168700
H	9.50396100	-0.05126000	-0.28452500
P	-4.21169700	1.51646600	-0.17294100
O	-5.51048000	1.95593400	0.61119800
H	-6.03017700	1.19521500	0.96997800
O	-4.55362300	-0.00401700	-0.71455500
O	-3.78521400	2.38242700	-1.28125200
P	-5.57811400	-1.10762700	-0.13652900
O	-4.69715600	-2.31881300	0.38911100
H	-4.16925200	-2.78707900	-0.27348200
O	-6.26850600	-1.54183800	-1.49980000
H	-6.99732200	-2.17211400	-1.40625200
O	-6.43275700	-0.55857800	0.94502200
H	7.61391500	-1.94985600	0.09093200

PP-pN6	$[C_{12}H_{20}N_4O_7P_2S]^{2+}$

2 1			
S	-1.37281300	1.97961100	-0.13693500
O	3.20545300	0.41374800	0.11951200
N	-2.66734200	-0.16535200	-0.31143300
N	-7.13513300	-0.09469500	1.10677900
N	-7.04941200	0.44168000	-1.19494800

N	-5.44140100	-1.13421900	2.31233200
C	-1.35544600	-0.53472700	-0.63312800
C	-3.76886400	-1.17421300	-0.29025300
C	-0.50762200	0.53424100	-0.58693500
C	0.96911300	0.56789500	-0.84969800
C	-5.14498100	-0.58546800	-0.13501900
C	-2.81796400	1.12057200	-0.02950800
C	-1.02209200	-1.94768900	-0.98353700
C	1.78951000	0.28094900	0.41475300
C	-5.91515200	-0.57341100	1.02052500
C	-5.82115200	-0.05467700	-1.24633200
C	-7.67599900	0.41929900	-0.00943900
C	-9.04909000	1.00196400	0.07435700
H	-3.52936500	-1.88878600	0.49581500
H	-3.72043400	-1.70856300	-1.23811100
H	1.25783300	1.54745600	-1.23582900
H	1.21844400	-0.16462900	-1.61892300
H	-3.75890700	1.57211000	0.23833000
H	-1.27087900	-2.63040800	-0.16761600
H	-1.55819500	-2.27262000	-1.87880400
H	0.04342900	-2.03986800	-1.18181900
H	1.58052400	-0.71237200	0.81665400
H	1.59090900	1.01718100	1.19130600

H	-5.33067800	-0.04608000	-2.21553900
H	-5.21049000	-2.13249500	2.25919800
H	-6.21137000	-1.03122400	2.98416700
H	-8.98056700	2.09414500	0.04599300
H	-9.64108200	0.69055000	-0.78726800
H	-9.53983900	0.70540700	0.99905900
P	4.06820800	-0.82643500	-0.40755100
O	4.32316300	-1.79601200	0.82620700
H	4.96431200	-1.42181000	1.47344600
O	5.48895100	-0.06190800	-0.65199800
O	3.56672600	-1.51590700	-1.60606600
P	6.56974700	0.46092600	0.44987100
O	6.36776400	2.02155000	0.58849700
H	6.50279100	2.54810500	-0.21203100
O	7.95062800	0.34400300	-0.31581100
H	8.24050300	-0.55275600	-0.53564800
O	6.39087200	-0.25355800	1.73509400
H	-4.62825900	-0.64012400	2.69647600

PP-pP39	$[C_{12}H_{20}N_4O_7P_2S]^{2+}$

2 1

S	1.46905200	1.22781100	1.93240700
O	-3.09830200	1.72819900	0.45192400
N	2.42887700	-0.42549800	0.30189600

N	6.67085700	0.60552500	-0.95556600
N	7.02687300	-1.19043900	0.56955400
N	4.62491200	1.07450300	-1.87360600
C	1.04577100	-0.56120800	0.14315900
C	3.41217800	-1.27176000	-0.46275700
C	0.36057000	0.28336500	0.96889000
C	-1.12511600	0.45867700	1.10446400
C	4.83224200	-0.81616900	-0.34729800
C	2.78823400	0.47118200	1.20466600
C	0.48775900	-1.54851200	-0.82874400
C	-1.64556700	1.58431600	0.21036200
C	5.36849200	0.28166000	-1.07105800
C	5.73608800	-1.50695900	0.44819600
C	7.43445000	-0.13033300	-0.14969400
C	8.88606600	0.24209000	-0.04167600
H	3.06858500	-1.27684700	-1.49789500
H	3.30915700	-2.28623600	-0.07707400
H	-1.38372500	0.68303200	2.14095800
H	-1.62268100	-0.47752500	0.84741400
H	3.81639300	0.68208200	1.45068700
H	0.74433100	-1.27980600	-1.85667800
H	0.86738600	-2.55355500	-0.63196800
H	-0.59708100	-1.57962200	-0.75532900

H	-1.47857100	1.39758600	-0.84868200
H	-1.23428800	2.55367500	0.47898100
H	5.39310500	-2.36896000	1.01602100
H	3.69163800	0.83081600	-2.15957300
H	5.11290000	1.76111200	-2.42804300
H	9.08352800	1.18124700	-0.55529600
H	9.17557200	0.32523400	1.00820100
H	9.50407400	-0.54423100	-0.48527100
P	-4.25141700	1.27696200	-0.46977100
O	-5.57672200	1.85436500	0.08603000
H	-6.20315900	1.14634200	0.42490500
O	-4.35539600	-0.30910000	-0.51290700
O	-3.89494700	1.78686500	-1.89693800
P	-5.53847300	-1.32108700	0.05106100
O	-4.78744100	-2.34699900	0.97802100
H	-4.72479000	-2.10416100	1.91420000
O	-5.80739200	-2.11279900	-1.27785100
H	-6.45962400	-2.82765800	-1.22944500
O	-6.60821300	-0.49008100	0.65767500
H	-4.43956600	1.50406700	-2.64862600

PP-pP45	$[C_{12}H_{20}N_4O_7P_2S]^{2+}$

2 1			
S	1.43889200	1.59735000	1.76451800

O	-3.12635600	2.01893700	0.17135300
N	2.32139800	-0.27704800	0.34387900
N	6.59498100	0.43167400	-1.01543400
N	6.88668600	-1.21840000	0.67919400
N	4.56072100	0.89631300	-1.96310000
C	0.93243800	-0.38093300	0.21480400
C	3.26312600	-1.24137800	-0.32586900
C	0.28715400	0.58299000	0.93501200
C	-1.18886300	0.83077800	1.05741000
C	4.70235200	-0.84050200	-0.25929100
C	2.72346700	0.70962500	1.12720600
C	0.32300000	-1.45714100	-0.62351800
C	-1.70911000	1.73656000	-0.06286200
C	5.27852600	0.15596800	-1.09046200
C	5.58234400	-1.48840300	0.59629400
C	7.33324900	-0.25408100	-0.14426700
C	8.80075400	0.06215200	-0.08029400
H	2.91638900	-1.33709300	-1.35570600
H	3.11655900	-2.20451000	0.16323500
H	-1.41008600	1.29922300	2.01812400
H	-1.71641200	-0.12436000	1.04029200
H	3.76080400	0.91221700	1.33863100
H	0.56126200	-1.32014700	-1.68154500

H	0.67567700	-2.44486300	-0.31864900
H	-0.76042900	-1.44273300	-0.52285600
H	-1.58663000	1.29090700	-1.04969400
H	-1.22543400	2.71054800	-0.05018800
H	5.20752300	-2.27441500	1.24799500
H	3.60808000	0.67988300	-2.20328400
H	5.06767100	1.50973600	-2.58234100
H	9.03529400	0.93451500	-0.68747900
H	9.10223000	0.23862400	0.95457800
H	9.37989200	-0.79203600	-0.44365900
P	-4.30872900	1.34325800	-0.61536600
O	-5.59271200	2.12364400	-0.14292200
H	-5.62855300	2.39355900	0.78632500
O	-4.41081800	-0.12161300	0.21710700
O	-4.21161800	1.11765500	-2.05956100
P	-5.09798000	-1.50713900	0.11519700
O	-4.70932900	-2.37238700	1.35230100
H	-5.07314000	-2.13351700	2.22042900
O	-4.50316400	-2.21734300	-1.13516500
H	-4.76088500	-3.13287100	-1.32834800
O	-6.62752700	-1.20460300	0.02433800
H	-7.25637500	-1.93796000	-0.07195600
